# *Salmonella* Typhimurium outer membrane protein A (OmpA) renders protection from nitrosative stress of macrophages by maintaining the stability of bacterial outer membrane

**DOI:** 10.1371/journal.ppat.1010708

**Published:** 2022-08-15

**Authors:** Atish Roy Chowdhury, Shivjee Sah, Umesh Varshney, Dipshikha Chakravortty

**Affiliations:** Department of Microbiology and Cell Biology, Indian Institute of Science, Bangalore, Karnataka, India; University of Pennsylvania, UNITED STATES

## Abstract

Bacterial porins are highly conserved outer membrane proteins used in the selective transport of charged molecules across the membrane. In addition to their significant contributions to the pathogenesis of Gram-negative bacteria, their role(s) in salmonellosis remains elusive. In this study, we investigated the role of outer membrane protein A (OmpA), one of the major outer membrane porins of *Salmonella*, in the pathogenesis of *Salmonella* Typhimurium (STM). Our study revealed that OmpA plays an important role in the intracellular virulence of *Salmonella*. An *ompA* deficient strain of *Salmonella* (STM *ΔompA*) showed compromised proliferation in macrophages. We found that the SPI-2 encoded virulence factors such as *sifA* and *ssaV* are downregulated in STM *ΔompA*. The poor colocalization of STM *ΔompA* with LAMP-1 showed that disruption of SCV facilitated its release into the cytosol of macrophages, where it was assaulted by reactive nitrogen intermediates (RNI). The enhanced recruitment of nitrotyrosine on the cytosolic population of STM *ΔompAΔsifA* and *ΔompAΔssaV* compared to STM *ΔsifA* and *ΔssaV* showed an additional role of OmpA in protecting the bacteria from host nitrosative stress. Further, we showed that the generation of greater redox burst could be responsible for enhanced sensitivity of STM *ΔompA* to the nitrosative stress. The expression of several other outer membrane porins such as *ompC*, *ompD*, and *ompF* was upregulated in STM *ΔompA*. We found that in the absence of *ompA*, the enhanced expression of *ompF* increased the outer membrane porosity of *Salmonella* and made it susceptible to *in vitro* and *in vivo* nitrosative stress. Our study illustrates a novel mechanism for the strategic utilization of OmpA by *Salmonella* to protect itself from the nitrosative stress of macrophages.

## Author summary

*Salmonella* Typhimurium majorly uses SPI-1 and SPI-2 encoded T3SS and virulence factors for thriving in the host macrophages. But the role of non-SPI genes in *Salmonella* pathogenesis remains unknown. This article illustrates a novel mechanism of how a non-SPI virulent protein, OmpA, helps *Salmonella* Typhimurium to survive in murine macrophages. Our data revealed that *Salmonella* lacking OmpA (STM *ΔompA*) is deficient in producing SPI-2 effector proteins and has a severe defect in maintaining the stability of its outer membrane. It is released into the cytosol of macrophages during infection after disrupting the SCV membrane. STM *ΔompA* was severely challenged with reactive nitrogen intermediates in the cytosol, which reduced their proliferation in macrophages. We further showed that the deletion of OmpA increased the expression of other larger porins (*ompC*, *ompD*, and *ompF*) on the surface of *Salmonella*. It was observed that the enhanced expression of OmpF in STM *ΔompA* increased the outer membrane permeability and made the bacteria more susceptible to *in vitro* and *in vivo* nitrosative stress. Altogether our study proposes new insights into the role of *Salmonella* OmpA as an essential virulence factor.

## Introduction

Bacterial porins are outer membrane-bound β barrel proteins with 8 to 24 anti-parallel β strands connected by extracellular loops. The porins are well known for their role in selective diffusion (of ions and solutes) and bacterial pathogenesis [1,2]. Outer membrane protein A (OmpA), one of the most abundant porins of the bacterial outer membrane, is extensively utilized by *Klebsiella pneumoniae* to prevent IL-8-dependent pro-inflammatory response in the airway epithelial A549 cells [3]. Deleting *ompC* and *ompF* from pathogenic *E*. *coli* impaired its invasion in bEnd.3 cells and reduced its virulence in a mouse model [4]. OprF, an OmpA ortholog in *Pseudomonas sp*., has been reported to function as a sensor of quorum signaling to induce virulence [5]. *E*. *coli* OmpW has been reported to play a role against phagocytosis and complement activation [6,7].

*Salmonella* Typhimurium is a member of the *Enterobacteriaceae* family, and it causes self-limiting gastroenteritis in humans. Its outer membrane is densely populated with many porins such as OmpA, OmpC, OmpD, and OmpF. Unlike OmpA, which tightly attaches the outer membrane to the underlying peptidoglycan layer with its periplasmic tail [8], other porins facilitate the transportation of charged ions [9]. The connection between the outer membrane porins and *Salmonella* pathogenesis remains elusive. Earlier, Heijden *et al*. proposed a mechanism that depicts OmpA and OmpC-dependent regulation of outer membrane permeability in *Salmonella* in response to H_2_O_2_ and antibiotic stresses [10].

In the current study, we have investigated the individual roles of OmpA, OmpC, OmpD, and OmpF in the pathogenesis of *Salmonella* Typhimurium with a significant focus on OmpA. We found that OmpA is required for the survival of wild-type *Salmonella* in macrophages. Deleting *ompA* downregulated the expression of SPI-2 effector *sifA*, which interrupted the intravacuolar niche of *Salmonella*. Our study further illustrates a novel mechanism on how intracellular *Salmonella* strategically utilizes OmpA to fight the nitrosative stress of macrophages by maintaining its outer membrane stability. STM *ΔompA* showed an increased expression of *ompC*, *ompD*, and *ompF*, which enhanced the permeability of the bacterial outer membrane. Furthermore, the upregulated expression of *ompF* escalated the susceptibility of STM *ΔompA* towards the *in vitro* and *in vivo* nitrosative stress.

## Results

### Deletion of OmpA impairs the intracellular proliferation of *Salmonella* Typhimurium in macrophages

The transcriptomics analyses of intracellular *Salmonella* indicated that during the early (4h), middle (8h), and late (12h) stages of infection in J774-A.1 macrophages and HeLa cells, the expression level of *ompA* is significantly upregulated (2 to 2.5 fold) in comparison with other major membrane porins such as *ompC*, *ompD*, and *ompF* [11,12]. To validate this observation, the transcript levels of *ompA*, *ompC*, *ompD*, and *ompF* from wild-type *Salmonella* growing in Luria-Bertani (LB) broth, acidic F media (pH = 5.4) [13], and RAW264.7 cells were measured by RT-qPCR (**S1A–S1D Fig**). *Salmonella* growing in *in vitro* conditions showed consistent time-dependent repression in expressing outer membrane porins (**S1A–S1D Fig**). However, there was a significant increase in the level of *ompA* during the late phase of infection in macrophages (**S1A–S1D Fig**), suggesting that intracellular *Salmonella* prefers *ompA* over other membrane-bound porins to survive inside the macrophages.

**Fig 1:**
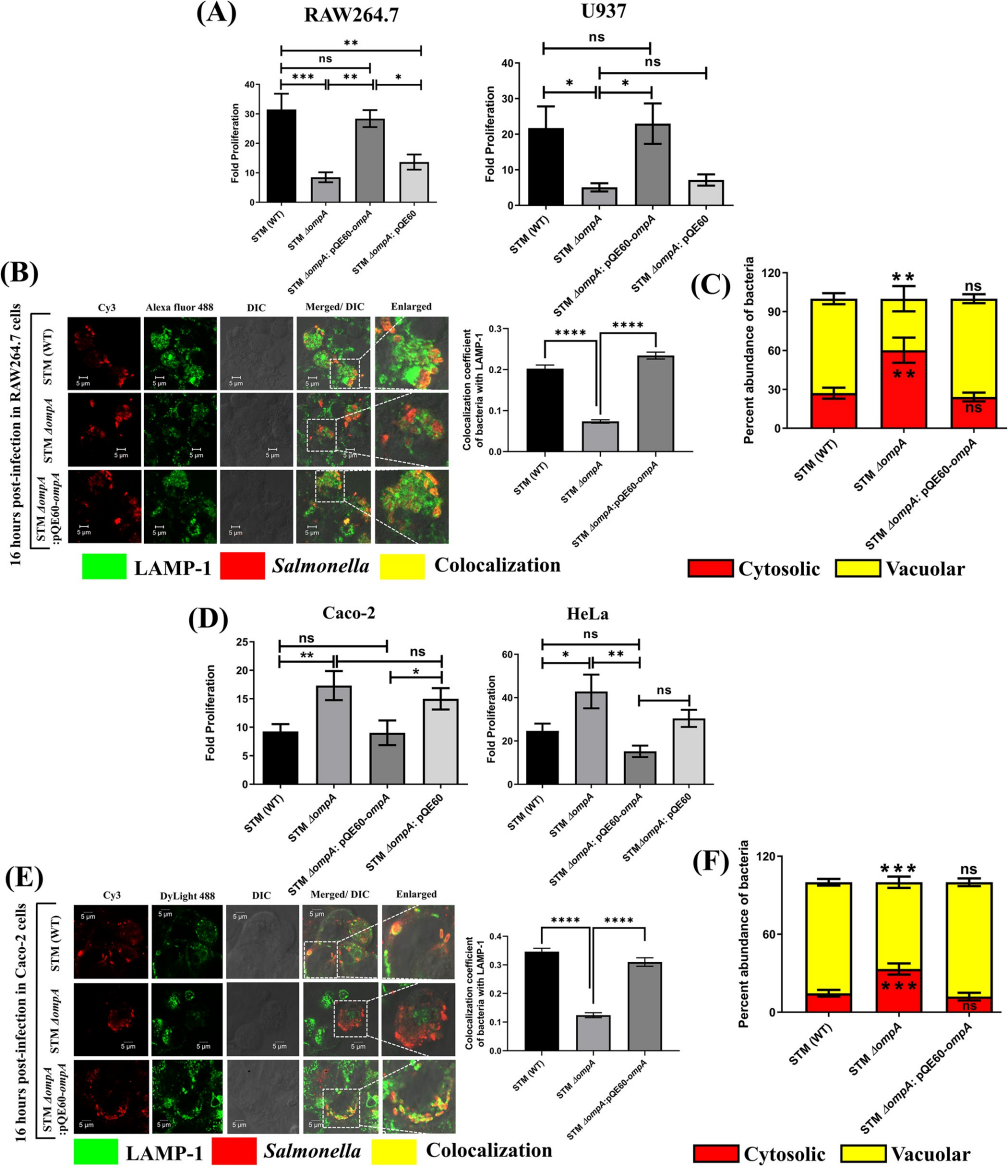
Deletion of OmpA impairs the intracellular proliferation of *Salmonella* Typhimurium in macrophages. (A) Fold proliferation of STM (WT), *ΔompA*, *ΔompA*: pQE60-*ompA*, & *ΔompA*: pQE60 in RAW264.7 and PMA activated U937 cells (MOI = 10) (n = 3, N = 3 for RAW 264.7 cells and n = 3, N = 2 for activated U937 cells). (B) Representative image of LAMP-1 recruitment on STM (WT), *ΔompA*, and *ΔompA*: pQE60-*ompA* (MOI = 20) in RAW264.7 cells. The colocalization coefficient of the bacteria with LAMP-1 has been represented in the form of a vertical bar graph. Scale bar = 5μm (n = 100, N = 4). (C) Chloroquine resistance assay of RAW264.7 cells infected with STM (WT), *ΔompA*, *ΔompA*: pQE60-*ompA* strains, respectively (n = 3, N = 2). (D) Fold proliferation of STM (WT), *ΔompA*, *ΔompA*: pQE60-*ompA*, & *ΔompA*: pQE60 in Caco-2 and HeLa cells (MOI = 10) (n = 3, N = 3). (E) Representative image of LAMP-1 recruitment on STM (WT), *ΔompA*, and *ΔompA*: pQE60-*ompA* (MOI of 20) in Caco-2 cells. The colocalization coefficient of the bacteria with LAMP-1 has been represented in the form of a vertical bar graph. Scale bar = 5μm (n = 100, N = 2). (F) Chloroquine resistance assay of Caco-2 cells infected with STM (WT), *ΔompA*, *ΔompA*: pQE60-*ompA* strains, respectively (n = 3, N = 2). Data are represented as mean ± SEM. *(P)* *< 0.05, *(P)* **< 0.005, *(P)* ***< 0.0005, *(P)* ****< 0.0001, ns = non-significant, (One-way ANOVA in A, B, D, E, and 2way ANOVA in C and F).

To understand the role of OmpA in *Salmonella* pathogenesis, we have generated an *ompA* deleted strain of *Salmonella* Typhimurium using the lambda red recombinase system [14]. We found that deleting *ompA* from *Salmonella* did not alter its *in vitro* growth in LB broth **(S2A and S2B Fig)**. It has been reported that Gram-negative bacteria such as *Pseudomonas aeruginosa* and *Legionella pneumophila* use outer membrane porins (OprF for *P*. *aeruginosa* and MOMP for *L*. *pneumophila*) to interact with complement proteins and facilitate phagocytosis [15,16]. We found that the deficiency of OmpA in *Salmonella* increased its phagocytosis by RAW264.7 and activated U937 cells (**S3A Fig**). The internalization of STM *ΔompA* (RAW264.7–0.003606 ± 0.0005081, U937- 0.00523 ± 0.00076)% by macrophages was significantly higher than STM (WT) (RAW264.7–0.001077 ± 0.0002955, U937- 0.00215 ± 0.00042)% and the complemented strain (RAW264.7–0.00199±0.00029, U937-0.00311 ± 0.00036)%. A significant increase in the phagocytosis of the complement-treated STM *ΔompA* (0.04964 ± 0.008098)% compared to complement-treated STM (WT) (0.02015 ± 0.00419)% and untreated STM *ΔompA* (0.02814 ± 0.004924)% confirmed the role of OmpA against complement recruitment and phagocytosis by host macrophages (**S3B Fig**). Adhesion of bacteria onto the host cell surface occurs before it enters the host cell [17]. Consistent with our previous observation, we found an enhanced attachment of STM *ΔompA* (4.446 ± 0.18) on RAW 264.7 cells compared to STM (WT) (3.00±0.19) and the complemented strain (3.54 ± 0.17) (**S3C Fig**). To establish the role of OmpA in the intracellular survival of *Salmonella*, gentamycin protection assay of STM (WT) and STM *ΔompA* was performed in RAW264.7 and activated U937 cells (**Fig 1A**). The intracellular proliferation of STM *ΔompA* (RAW264.7–8.486 ± 1.697, U937- 5.075 ± 1.157) was significantly attenuated in macrophages when compared with its wild-type (RAW264.7–31.5 ± 5.347, U937- 21.71 ± 6.094) and complemented counterparts (RAW264.7–28.39 ± 2.88, U937- 22.97 ± 5.678) (**Fig 1A**). We concluded that in *Salmonella* Typhimurium, OmpA serves dual functions to protect the bacteria from phagocytosis and then help in its survival within macrophages. After entering the host cell, *Salmonella* resides within a modified phagosomal compartment called *Salmonella* containing vacuole (SCV) [18]. The intracellular life and proliferation of *Salmonella* depend on the stability and integrity of SCV. *Salmonella* recruits a plethora of host proteins such as LAMP-1, Rab7, and vATPase to maintain the sustainability of SCV [19,20]. Since we observed an attenuated intracellular proliferation of STM *ΔompA* in macrophages, we decided to check the intracellular niche of the bacteria using LAMP-1 as a marker of SCV (**Fig 1B**). It was observed that the colocalization coefficient of STM *ΔompA* with LAMP-1 is less compared to STM (WT) in RAW264.7 cells (**Fig 1B**). The colocalization of bacteria with LAMP-1 was recovered in the complemented strain. This observation suggested a significant loss of the SCV membrane from STM *ΔompA* during infection in macrophages (**Fig 1B**). This result was further supported by the chloroquine resistance assay in macrophages (**Fig 1C**). The protonated chloroquine cannot exit the SCV and kills the vacuolar population of *Salmonella*. So the cytosolic bacteria survive. Our data demonstrated that at 16 h post-infection, the cytosolic abundance of STM *ΔompA* was more than the wild-type and complemented strains in RAW264.7 cells (**Fig 1C**). *Salmonella* invades M cells of the Peyer’s patches in the small intestine with the help of the SPI-1 encoded type III secretion system (T3SS). The cooperative activity of SPI-1 T3SS and SPI-4 encoded adhesin SiiE helps *Salmonella* to infect polarized epithelial cells [21]. Therefore, we decided to study the role of *Salmonella* OmpA in its invasion of the epithelial cells (**S4A Fig**). Compared to the wild-type bacteria (Caco-2- 0.006199 ± 0.001562, HeLa- 0.006352 ± 0.000955)%, STM *ΔompA* (Caco-2- 0.00239 ± 0.00049, HeLa- 0.002299 ± 0.0003608)% exhibited an attenuated invasion of epithelial cells (**S4A Fig**). The bacterial invasion was recovered upon complementation of OmpA (Caco-2- 0.004011 ± 0.00092, HeLa- 0.00666 ± 0.00175)% (**S4A Fig**). To further validate this observation, we carried out an adhesion assay using HeLa cells (**S4B Fig**). STM *ΔompA* (3.893 ± 0.4042) showed reduced attachment to the surface of HeLa cells compared to the wild-type (9.471 ± 0.9633) and the complemented strains (7.652 ± 0.8699) (**S4B Fig**). This observation is consistent with the result obtained from the invasion assay and shows the utilization of OmpA by *Salmonella* as an important adhesion and invasion tool for the epithelial cells. Further, we investigated the role of OmpA in maintaining the intracellular life of bacteria in epithelial cells (**Fig 1D**). Contrary to the macrophages, we found that STM *ΔompA* (Caco-2- 17.32 ± 2.55, HeLa- 42.85 ± 7.79) is hyperproliferating in epithelial cells compared to the wild-type (Caco-2- 9.26 ± 1.28, HeLa- 24.7 ± 3.3) and the complemented strain (Caco-2- 9.02 ± 2.17, HeLa- 15.21 ± 2.66) (**Fig 1D**). Interestingly, STM *ΔompA* showed a reduced colocalization with LAMP-1 compared to the wild-type and the complemented strains in Caco-2 cells suggesting a disruption of the SCV membrane (**Fig 1E**). Chloroquine resistance assay showed that the cytosolic population of STM *ΔompA* was more compared to the wild-type and the complemented strains in Caco-2 cells **(Fig 1F)**. Altogether, our data suggest that in the absence of OmpA, *Salmonella* starts losing LAMP-1 and eventually comes into the cytosol of host cells from the SCV. In the cytosol of macrophages, the mutant bacteria is killed, whereas it can survive well in the cytosol of epithelial cells.

### Deficiency of OmpA downregulates the expression of SPI-2 effector proteins in *Salmonella*

**Fig 2:**
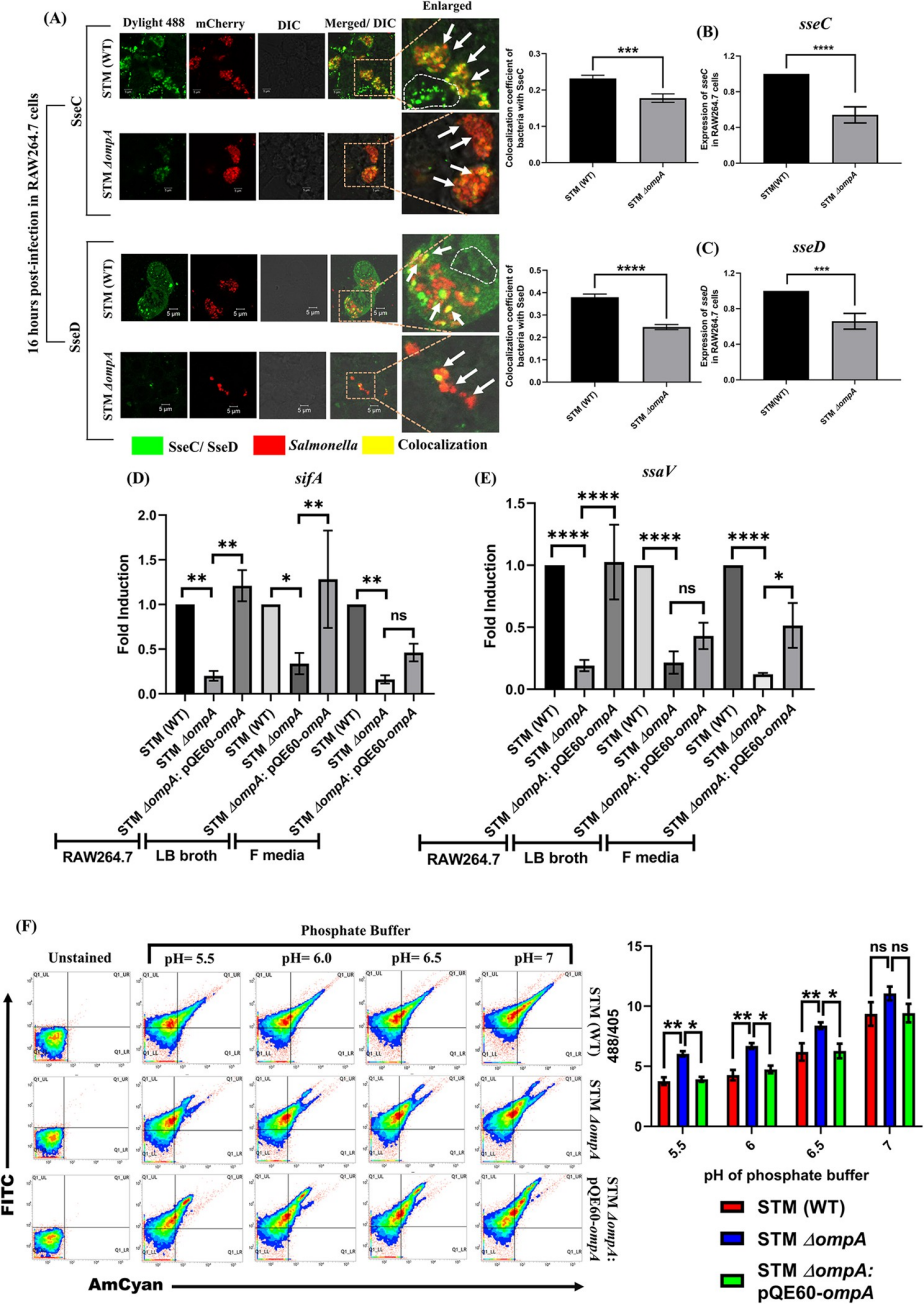
Deficiency of OmpA downregulates the expression of SPI-2 effector proteins in *Salmonella*. (A) Representative image of SseC/ SseD recruitment on STM (WT) and *ΔompA* expressing RFP (MOI 20) in RAW264.7 cells. The colocalization coefficient of bacteria with SseC and SseD were represented as vertical bar graphs. Scale bar = 5μm, (n = 50, N = 3). Quantification of the expression of (B) *sseC* and (C) *sseD* in STM (WT) and *ΔompA* growing intracellularly in RAW264.7 cells by RT-qPCR (n = 3, N = 3). The expression profile of (D) *sifA* and (E) *ssaV* in STM (WT), *ΔompA*, and *ΔompA*-pQE60-*ompA* growing in RAW264.7 cells, LB broth, and acidic F media, respectively (n = 3, N = 3). (F) Studying the intracellular acidification of BCECF-AM stained STM (WT), *ΔompA*, and *ΔompA*: pQE60-*ompA* in phosphate buffer of pH 5.5, 6, 6.5, and 7, respectively, using 20 μM of BCECF-AM by flow cytometry. The ratio of BCECF-AM (MFI) at 488 and 405 nm were represented as a vertical bar graph (n = 4, N = 3). Data are represented as mean ± SEM. *(P)* *< 0.05, *(P)* **< 0.005, *(P)* ***< 0.0005, *(P)* ****< 0.0001, ns = non-significant, (Unpaired stuedent’s *t* test in A, B, C and one-way ANOVA in D, E, F).

The pH inside the SCV is acidic (pH = 5.4) in comparison with the cytosol (pH = 7.4) of the macrophages [22], [23]. The wild-type *Salmonella* senses the acidic environment of the SCV with the help of two-component systems and activates the expression of SPI-2 genes to establish an actively replicating niche [22,24–26]. SPI-2 codes for a T3SS and several effector proteins, which are eventually localized either on the SCV or in different subcellular organelles, including the nucleus, Golgi, ER, and cytosol [27–29]. Since the cytosolic population of STM *ΔompA* lacks an intact SCV membrane, we hypothesized that the interruption in the assembly of the SPI-2-T3SS needle would hamper the biogenesis and secretion of SPI-2 effectors into the host cytosol. Interestingly, we found a marked reduction in the accumulation and secretion (the area of the infected macrophage demarcated with a dotted line for the wild-type *Salmonella*) of two such SPI-2 effectors, SseC (**Fig 2A**) and SseD (**Fig 2A**) on or from the surface of STM *ΔompA* into the host cytosol. In continuation of this observation, we found an attenuated expression of *sseC* (**Fig 2B**) and *sseD* (**Fig 2C**) genes in intracellularly growing STM *ΔompA* compared to the wild-type bacteria. To find out whether the reduced expression of *sseC* and *sseD* is because of the lack of acidification of STM *ΔompA* in the cytosol of macrophages, we measured the level of *sseC* and *sseD* transcripts in wild-type and OmpA deficient *Salmonella* growing in LB broth and acidic F media **(S5A–S5D Fig)**. Surprisingly, it was found that STM *ΔompA* is deficient in expressing *sseC* and *sseD* under *in vitro* growth conditions, suggesting that the deletion of *ompA* might have a role in suppressing the expression of SPI-2 encoded virulent genes. To further validate this hypothesis, we quantified the expression of two other SPI-2 effectors, *sifA* (**Fig 2D**) and *ssaV* (**Fig 2E**), in STM (WT) and *ΔompA*. We found that the expression of *sifA* and *ssaV* was significantly attenuated in STM *ΔompA* growing intracellularly (**Fig 2D and 2E**). Complementing *ompA* recovered the expression of these SPI-2 genes. In addition, the reduced expression of *sifA* and *ssaV* in STM *ΔompA* growing in LB broth and F media confirmed that deleting OmpA directly downregulates the expression of SPI-2 genes. The inability of STM *ΔompA* to induce the expression of *sifA*, *ssaV*, *sseC*, and *sseD* in the SPI-2 inducing acidic F media suggested an important role of OmpA in the acidification of the cytosol of *Salmonella*. To determine the degree of acidification upon altering the pH of the surrounding media, we used a pH-sensitive dye BCECF-AM. We observed a higher 488 nm/ 405 nm ratio of STM *ΔompA* labeled with BCECF-AM when resuspended in phosphate buffer of acidic pH (5.5, 6, and 6.5) compared to STM (WT) and the complemented strain (**Fig 2F**). This result suggests reduced acidification of the cytosol of STM *ΔompA* compared to STM (WT) and STM *ΔompA*: pQE60-*ompA* even when they are present in the same acidic environment (**Fig 2F**). Surprisingly when all these strains were incubated in the phosphate buffer of pH = 7, we found a comparable 488 nm/ 405 nm ratio of BCECF-AM, unveiling an uncharacterized novel role of OmpA in the acidification of the cytosol of *S*. Typhimurium in response to extracellular acidic stress. Earlier it has been reported that the deletion of *sifA* increases the cytosolic abundance of *Salmonella* in both epithelial and macrophage cells [30]. Hence, we concluded that downregulation of the expression of SPI-2 effectors in the absence of OmpA might release the STM *ΔompA* from SCV into the cytosol of macrophages and epithelial cells.

### In addition to *sifA* and *ssaV*, *Salmonella* uses OmpA to combat the nitrosative stress of macrophages

**Fig 3:**
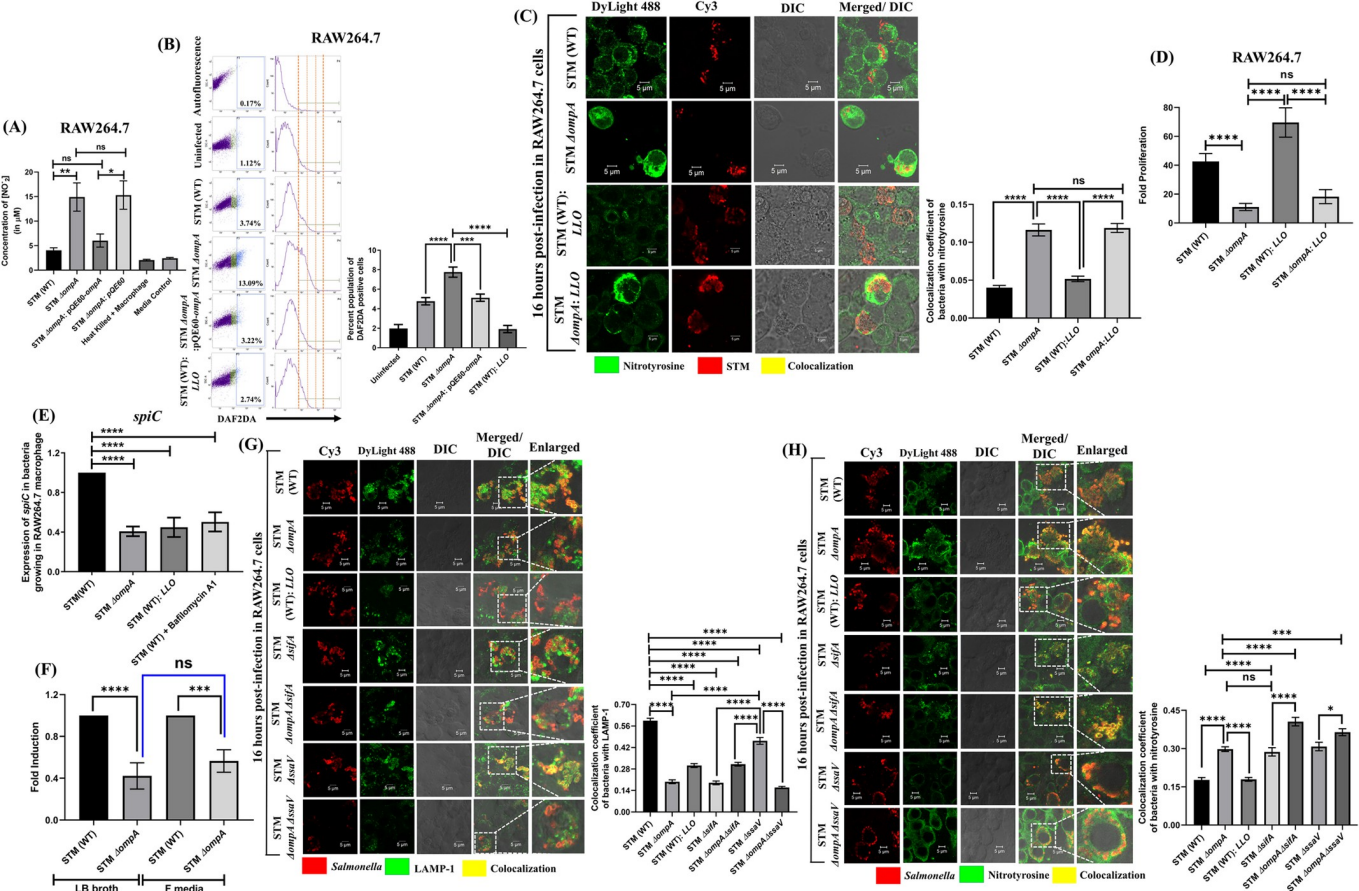
In addition to *sifA* and *ssaV*, *Salmonella* uses OmpA to combat the nitrosative stress of macrophages. (A) Estimating the extracellular nitrite from the culture supernatant of RAW264.7 cells infected with STM (WT), *ΔompA*, *ΔompA*: pQE60-*ompA*, *ΔompA*: pQE60, & heat-killed bacteria (MOI = 10) respectively by Griess assay (n = 3, N = 5). (B) Representative dot plots (SSC-A vs. DAF-2 DA) and histograms (Count vs. DAF-2 DA) of RAW264.7 cells infected with STM (WT), *ΔompA*, *ΔompA*: pQE60-*ompA*, and (WT): *LLO* (MOI 10) to estimate the level of intracellular nitric oxide (NO) using DAF-2 DA (5 μM). The percent population of DAF-2 DA positive cells was represented in a vertical bar graph (n≥3, N = 6). (C) Immunofluorescence image of RAW264.7 cells infected with STM (WT), *ΔompA*, (WT): *LLO*, and *ΔompA*: *LLO* at MOI 20. The colocalization coefficient of bacteria with nitrotyrosine was represented as a vertical bar graph. Scale bar = 5μm (n = 50, N = 3). (D) Fold proliferation of STM (WT), *ΔompA*, (WT): *LLO*, and *ΔompA*: *LLO* in RAW264.7 cells (n≥3, N = 2). (E) Quantifying the transcript-level expression of *spiC* from RAW264.7 cells infected with STM (WT), *ΔompA* & (WT): *LLO* at MOI 50. STM (WT) infected RAW264.7 cells treated with bafilomycin A (50 nM) were used as a control (n = 3, N = 3). (F) Estimating the expression of *spiC* by RT-qPCR in STM (WT) and *ΔompA* growing in LB broth and acidic F media (n = 3, N = 5). (G) Representative image of LAMP-1 recruitment on STM (WT), *ΔompA*, (WT): *LLO*, *ΔsifA*, *ΔompAΔsifA*, *ΔssaV*, *ΔompAΔssaV* (MOI = 20) in RAW264.7 cells. The colocalization coefficient of the bacteria with LAMP-1 was represented as a vertical bar graph. Scale bar = 5μm (n = 80, N = 3). (H) Representative image of RAW264.7 cells infected with STM (WT), *ΔompA*, (WT): *LLO*, *ΔsifA*, *ΔompAΔsifA*, *ΔssaV*, *ΔompAΔssaV* (MOI = 20) to visualize the recruitment of nitrotyrosine on the bacteria. The vertical bar graph depicts the colocalization coefficient of bacteria with intracellular nitrotyrosine. Scale bar = 5μm, (n = 100, N = 3). Data are represented as mean ± SEM. *(P)* *< 0.05, *(P)* **< 0.005, *(P)* ***< 0.0005, *(P)* ****< 0.0001, ns = non-significant, (One-way ANOVA).

During the early and late stages of infection in macrophages, intracellular *Salmonella* is periodically challenged with oxidative (ROS) and nitrosative stresses (RNI) [31,32]. The SCV membrane protects the vacuolar niche of wild-type *Salmonella* from these potential threats present in the cytosol of macrophages [30,33]. Our study demonstrated that STM *ΔompA* is released from SCV into the cytosol of macrophages and epithelial cells. Therefore, we decided to study the oxidative and nitrosative response of the host cells infected with STM *ΔompA*. We quantified the level of extracellular NO produced by the infected macrophages by Griess assay (**Fig 3A**). It was found that during the late stage of infection, nitrite accumulation in the culture supernatant of RAW264.7 cells infected with STM *ΔompA* was significantly higher compared to the wild-type. This heightened NO response was not seen when the macrophages were infected with the complemented bacteria (**Fig 3A**). This result was further validated by quantifying the level of intracellular NO using DAF2-DA (**Fig 3B**). Only (4.77 ± 0.37)% of wild-type bacteria-infected macrophages produced NO **(Fig 3B)**, which was increased to (7.76 ± 0.52)% when the cells were infected with STM *ΔompA* (**Fig 3B**). The percentage of RAW264.7 cells producing NO after being infected with complemented strain (5.13 ± 0.37)% was comparable to the STM (WT) **(Fig 3B)**. *Listeria monocytogenes* produce listeriolysin O (LLO), a pore-forming toxin to degrade the vacuolar membrane to escape lysosomal fusion [34]. Surprisingly, STM (WT) expressing LLO showed poor induction of NO (1.91 ± 0.36)% (**Fig 3B**) during their stay in the cytosol of macrophages. Further, we investigated the intra and extracellular oxidative response of the macrophages upon *Salmonella* infection by H_2_DCFDA staining (**S6A and S6B Fig**) and phenol red assay (**S6C Fig**), respectively. We did not find any considerable change in the level of ROS produced by the macrophages infected with wild-type (2.157 ± 0.1611)%, knockout (2.192 ± 0.2955)%, or the complemented strains (2.61 ± 0.2244)% of *Salmonella* (**S6A and S6B Fig**). Consistent with this, no significant change was observed in the level of H_2_O_2_ accumulated in the supernatant of the infected cells (**S6C Fig**). The NO produced from the cellular pool of L- arginine by inducible nitric oxide synthase (iNOS) is further oxidized into NO-adducts (NONOates, peroxynitrite, nitrite), which have higher oxidation potential and bactericidal activity [35]. The damage caused by peroxynitrite (ONOO^-^) can be monitored microscopically by studying the recruitment of nitrotyrosine on the surface of intracellular bacteria (**Fig 3C**). While infecting the RAW264.7 cells, STM *ΔompA* showed greater colocalization with nitrotyrosine than the wild-type bacteria (**Fig 3C**), indicating the damage caused by peroxynitrites. Interestingly, the cytosolic population of STM (WT): *LLO* showed poor colocalization with nitrotyrosine, suggesting a possible OmpA-dependent protective mechanism of *Salmonella* from the RNI (**Fig 3C**). Conversely, the greater recruitment of nitrotyrosine on STM *ΔompA*: *LLO* (**Fig 3C**) showed that the OmpA-dependent protection of STM (WT): *LLO* from RNI is independent of LLO. Consistent with this observation, we found that the intracellular survival of STM (WT): *LLO* in macrophages was better than STM *ΔompA* and STM *ΔompA*: *LLO* (**Fig 3D**). *Salmonella* utilizes SPI-2 encoded virulence factor SpiC to downregulate the activity of iNOS in a SOCS-3-dependent manner [36,37]. Hence, we quantified the level of *spiC* in *Salmonella* in the absence of *ompA*. The reduced expression of *spiC* and increased NO response in macrophages infected with STM *ΔompA* indicated that wild-type *Salmonella* doesn’t require OmpA to suppress the nitrosative stress **(Fig 3E)**. Macrophages infected with either STM (WT): *LLO* (deficient in expressing *spiC* for its cytosolic localization) or STM (WT) under bafilomycin A1 treatment afterward (inhibiting the acidification of SCV) were used as positive controls for this phenotype **(Fig 3E)**. To validate this observation, the promoter activity of *spiC* was measured in STM (WT) and *ΔompA* growing in macrophages (**S7A Fig**) by beta-galactosidase assay. Inside the macrophages, a significant drop in the activity of the *spiC* promoter was observed in STM *ΔompA* (**S7A Fig**), which disappeared when the bacteria were grown under *in vitro* conditions **(S7B Fig)**. On the contrary, STM *ΔompA* showed severely reduced expression of *spiC* in LB and F media **(Fig 3F)**. The complementation of STM *ΔompA* with pHG86: *spiC-lacZ* plasmid might be responsible for overriding the attenuation of *spiC* during its *in vitro* growth. Deletion of SPI-2 effectors makes *Salmonella* susceptible to intracellular nitrosative and oxidative stresses [33,35,38]. Earlier, we have found that STM *ΔompA* is deficient in expressing SPI-2 effectors *sifA* and *ssaV*. To establish the specific role of OmpA in protecting *Salmonella* from the damage caused by nitrosative stress, we infected macrophages with STM *ΔsifA*, *ΔssaV*, *ΔompAΔsifA*, and *ΔompAΔssaV*. We observed that STM *ΔsifA* and *ΔompAΔsifA* came into the cytosol of macrophages after exiting the SCV **(Fig 3G)**. Deleting *ompA* from STM *ΔssaV* reduced the colocalization of STM *ΔompAΔssaV* with LAMP-1 and resulted in their release into the cytosol **(Fig 3G)**. Simultaneously, we studied the recruitment of nitrotyrosine on these bacterial strains **(Fig 3H)**. Compared to the wild-type bacteria, STM *ΔompA*, *ΔsifA*, and *ΔssaV* showed enhanced recruitment of nitrotyrosine **(Fig 3H)**. The higher colocalization of nitrotyrosine with STM *ΔompAΔsifA* and *ΔompAΔssaV* compared to STM *ΔsifA*, and *ΔssaV* suggested a SPI-2 independent additional role of OmpA in protecting the bacteria from nitrosative stress of macrophages **(Fig 3H)**. The attenuated intracellular proliferation of STM *ΔompAΔsifA* and *ΔompAΔssaV* compared to STM *ΔsifA*, and *ΔssaV* supported this observation **(S8A Fig)**. However, compared to STM (WT), the higher and indistinguishable level of intracellular NO induced by STM *ΔompA*, *ΔsifA*, *ΔompAΔsifA*, *ΔssaV*, and *ΔompAΔssaV* **(S8B Fig)** suggested that the increased NO response of macrophages upon STM *ΔompA* infection depends on the attenuated expression of SPI-2 genes. To find the reason for the hyperproliferation of STM *ΔompA* in epithelial cells, the nitrosative burst of infected Caco-2 cells was checked **(S9A Fig)**. The inability of STM *ΔompA* to induce a higher NO response compared to the wild-type or the complemented strain supports its overgrowth in the cytosol of Caco-2 cells.

### Modulating the iNOS activity by using a specific inhibitor or activator determines the fate of STM *ΔompA* in *in vitro* and *in vivo* infection models

**Fig 4:**
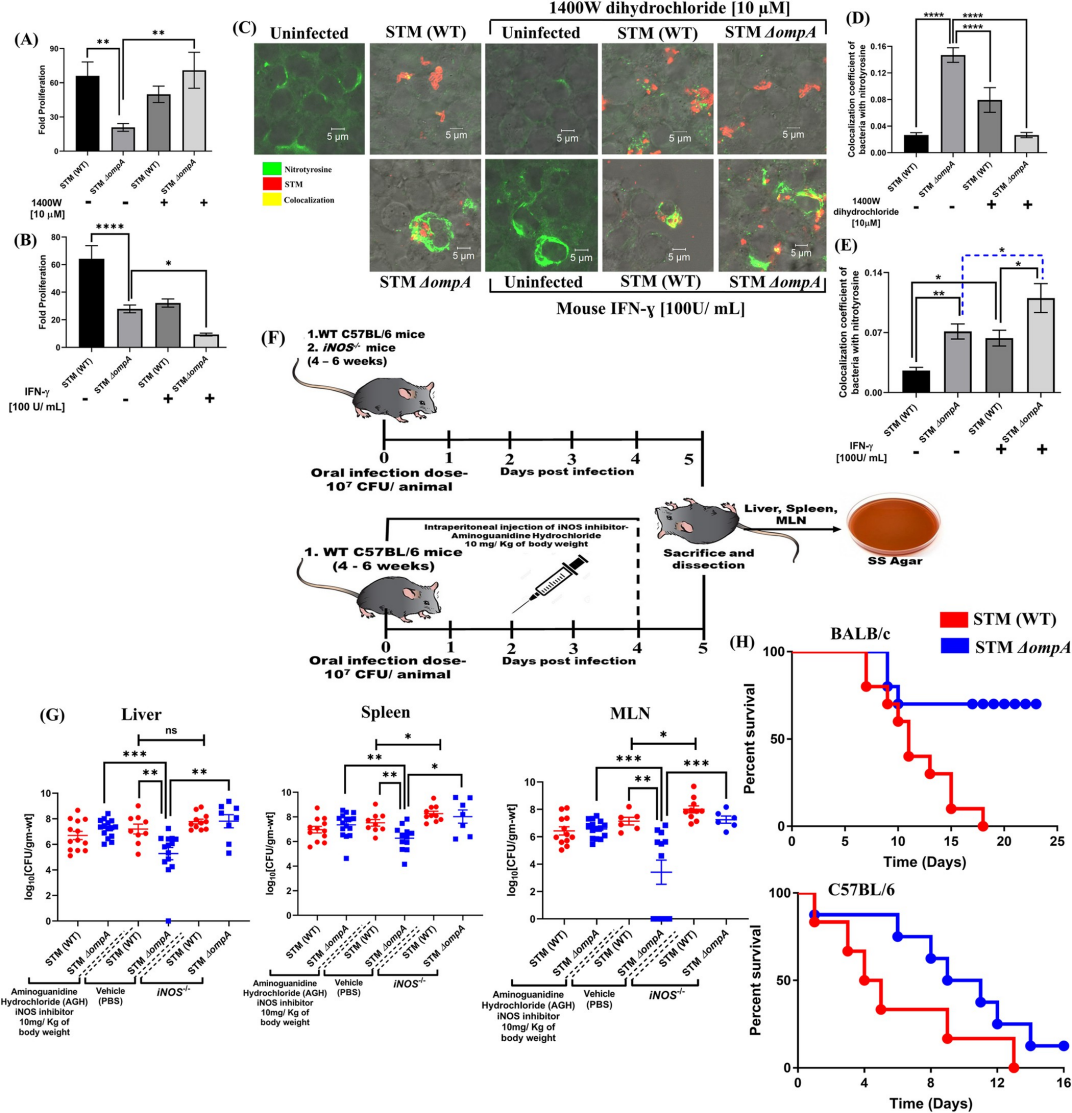
Modulating the activity of iNOS by using a specific inhibitor or activator determines the fate of STM *ΔompA* in *in vitro* and *in vivo* infection models. Intracellular survival of STM (WT) and *ΔompA* (MOI = 10) in RAW264.7 cells (16 hours post-infection) in the presence and absence of iNOS (A) inhibitor- 1400W dihydrochloride (10 μM) and (B) activator- mouse IFN-ɣ (100U/ Ml) (n = 3, N = 3). (C) Immunofluorescence image of RAW264.7 cells infected with STM (WT) and *ΔompA* (MOI = 20) in the presence and absence of (D) 1400W dihydrochloride and (E) mouse IFN-ɣ. The colocalization coefficient of bacteria with nitrotyrosine was represented in vertical bar graphs (n≥ 50, N = 2). (F) The schematic representation of the experimental strategy for studying the *in vivo* pathogenesis of STM (WT) and *ΔompA*. (G) Enumerating the bacterial load in the liver, spleen, and MLN of C57BL/6 mice orally gavaged with STM (WT) and *ΔompA* in the presence and absence of iNOS inhibitor aminoguanidine hydrochloride (10mg/ kg of body weight). The bacterial load from the liver, spleen, and MLN of *iNOS*^*-/-*^ C57BL/6 mice were also quantified. The log_10_(CFU/ gm-wt.) for each CFU obtained after plating was plotted (n = 5, N = 3). (H) Studying the survival BALB/c and C57BL/6 mice infected with a lethal dose of STM (WT) and *ΔompA*. The survival study was monitored till the death of all the wild-type infected mice (n = 10). Data are represented as mean ± SEM. *(P)* *< 0.05, *(P)* **< 0.005, *(P)* ***< 0.0005, *(P)* ****< 0.0001, ns = non-significant, (One-way ANOVA in A, B, D, E and Mann-Whitney *U* test in G).

To substantiate the protective role of *Salmonella* OmpA against the nitrosative stress, we treated the macrophages with an irreversible inhibitor (1400W dihydrochloride at 10 μM concentration) (**Fig 4A, 4C, and 4D**) and an activator (mouse IFN-ɣ at 100 U/ mL concentration) of iNOS (**Fig 4B, 4C, and 4E**). Inhibiting the activity of iNOS using 1400W completely restored the intracellular proliferation of STM *ΔompA* (70.97 ± 15.75) compared to the untreated control (20.86 ± 3.387) (**Fig 4A**). Consistent with this finding, STM *ΔompA* showed poor colocalization with nitrotyrosine upon 1400W treatment (**Fig 4C and 4D**). Augmenting the iNOS activity using mouse IFN-ɣ hindered the intracellular proliferation of STM *ΔompA* more efficiently (reduction of fold proliferation from 27.91 ± 2.791 to 9.295 ± 1.004–3-fold) than the wild-type bacteria (reduction of fold proliferation from 64.27 ± 9.483 to 32.15 ± 2.967–2-fold) (**Fig 4B**). Concomitantly, STM *ΔompA* showed enhanced colocalization with nitrotyrosine in IFN-ɣ treated macrophages, resulting in their attenuated intracellular proliferation (**Fig 4C and 4E**). The reduced CFU burden of *ompA* mutant compared to the wild-type *Salmonella* in the liver, spleen, and MLN of C57BL/6 mice strongly supports the role of OmpA in bacterial pathogenesis (**Fig 4F and 4G**). However, the attenuated burden of *ompA* mutant was not seen in the liver, spleen, and MLN of aminoguanidine hydrochloride (AGH) treated and *iNOS*^*-/-*^ C57BL/6 mouse [39]. Compared to the PBS-treated mice, the restoration of higher STM *ΔompA* burden in AGH treated and *iNOS*^*-/-*^ C57BL/6 mice concomitantly confirmed the role of OmpA in protecting *Salmonella* from *in vivo* nitrosative stress (**Fig 4G**). To observe the role of *ompA* during *in vivo* infection of *Salmonella* Typhimurium, we challenged 4–6 weeks old adult BALB/c and C57BL/6 mice (**Fig 4H**) with a lethal dose (10^8^ CFU of bacteria/ animal) of wild-type and knockout strains. Almost 80% of BALB/c mice infected with STM *ΔompA* survived compared to the group infected with wild-type bacteria (**Fig 4H**). On the other side, the C57BL/6 mice infected with STM *ΔompA* showed better survival and retarded death than those infected with the wild-type bacteria, suggesting a critical role of *ompA* in *Salmonella-*mediated mortality.

### OmpA-dependent regulation of outer membrane permeability in *Salmonella* controls cytoplasmic redox homeostasis in response to *in vitro* nitrosative stress

**Fig 5:**
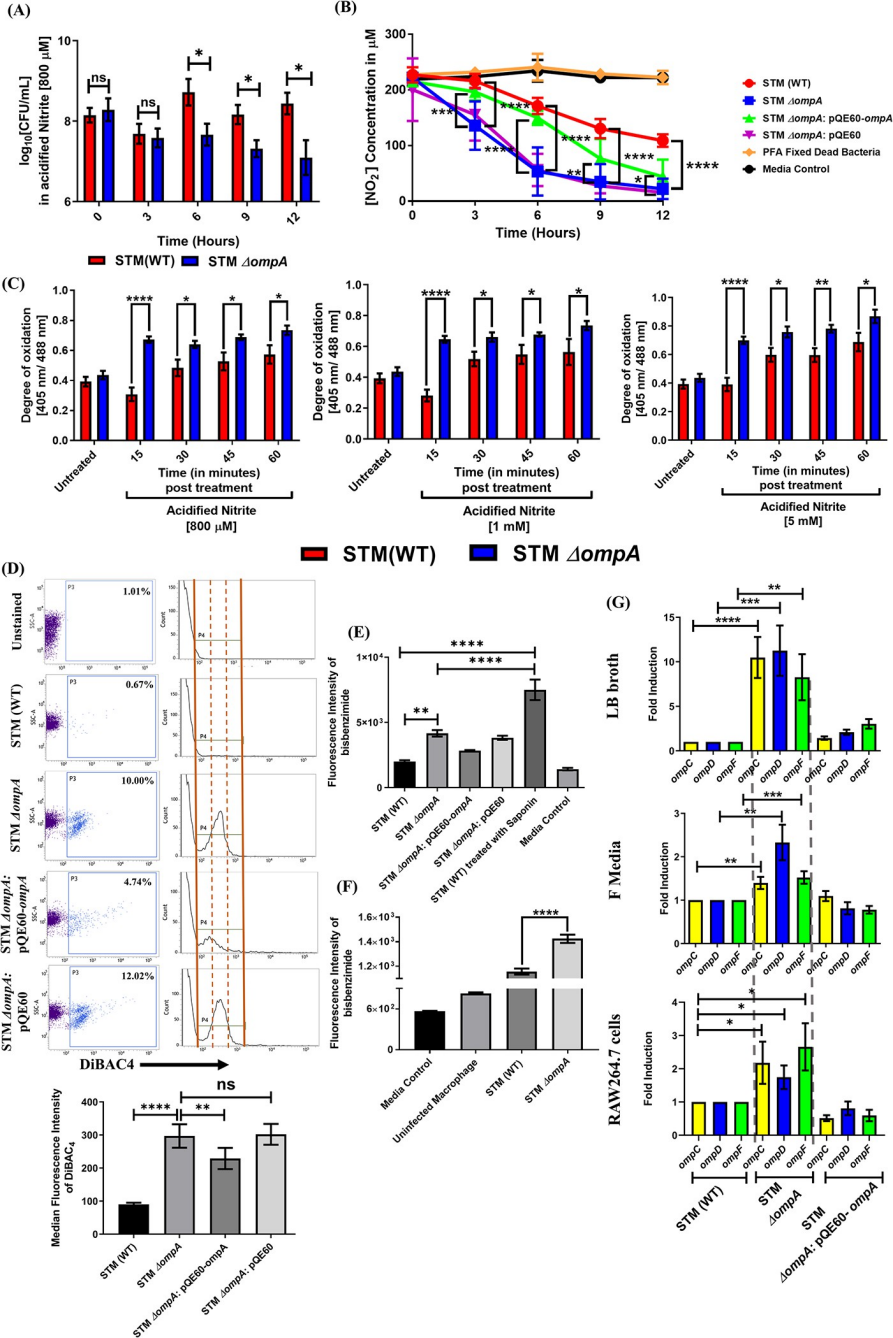
OmpA-dependent regulation of outer membrane permeability in *Salmonella* controls cytoplasmic redox homeostasis in response to *in vitro* nitrosative stress. (A) Time-dependent *in vitro* death kinetics of STM (WT) and *ΔompA* in the presence of acidified nitrite (Nitrite concentration 800 μM in PBS of pH 5.4). Data are represented as mean ± SEM (N = 5). (B) *In vitro* nitrite uptake assay of STM (WT), *ΔompA*, *ΔompA*: pQE60-*ompA*, *ΔompA*: pQE60, & PFA fixed dead bacteria (n = 3, N = 4). (C) Time-dependent measurement of redox homeostasis of STM (WT) and *ΔompA* harboring pQE60-Grx1-roGFP2 in response to varying concentrations of acidified nitrite. Median fluorescence intensities of Grx1-roGFP2 at 405nm and 488nm for the FITC positive population were used to obtain the 405/ 488 ratio (n = 3, N = 3). (D) The dot plots (SSC-A vs. DiBAC_4_) and histograms (Count vs. DiBAC_4_) representing the outer membrane porosity of STM (WT), *ΔompA*, *ΔompA*: pQE60-*ompA*, & *ΔompA*: pQE60 growing in acidic F media (12 hours post-inoculation). The median fluorescence intensity of DiBAC_4_ (final concentration- 1 μg/ mL) has been represented as a vertical bar graph (n = 3, N = 2). (E) Measurement of outer membrane porosity of STM (WT), *ΔompA*, *ΔompA*: pQE60-*ompA* & *ΔompA*: pQE60 in acidic F media (12 hours post-inoculation) using bisbenzimide (excitation- 346 nm and emission- 460 nm), (final concentration- 1 μg/ mL), (n = 8, N = 3). (F) Measuring the outer membrane porosity of STM (WT) and *ΔompA* isolated from RAW264.7 cells at 12 hours post-infection by bisbenzimide (Sigma) (final concentration- 1 μg/ mL), (n = 6, N = 3). (G) Quantifying the expression profile of larger porins (*OmpC*, *OmpD*, *OmpF*) by RT-qPCR in STM (WT), *ΔompA*, & *ΔompA*: pQE60-*ompA* growing in LB broth, F media, and RAW264.7 cells (MOI 50) (12 hours post-inoculation), (n = 3, N = 3). Data are represented as mean ± SEM. *(P)* *< 0.05, *(P)* **< 0.005, *(P)* ***< 0.0005, *(P)* ****< 0.0001, ns = non-significant, (Unpaired student’s *t-*test in A, B, C, G and one-way ANOVA in D, E, F).

In a mildly acidic environment (pH = 5–5.5), NaNO_2_ dissociates to form nitrous acid (HNO_2_), which undergoes a dismutation reaction upon oxidation and generates a wide range of reactive nitrogen intermediates (RNI) such as NO_2_, N_2_O_3_, and NO, which kill the bacteria by causing damage to nucleic acids, proteins and lipids [40,41]. To investigate the role of *Salmonella* OmpA against *in vitro* nitrosative stress, we checked the *in vitro* sensitivity of the wild-type and mutant strains in the presence of varying concentrations (0–5 mM) of H_2_O_2_ (**S6D Fig**), NaNO_2_ (**S6E Fig**), and a combination of the two for 12 h (**S6F Fig**) by CFU counting and resazurin assay. Compared to the wild-type bacteria, STM *ΔompA* did not show any significant difference in viability when exposed to peroxide (**S6D Fig**). However, the knockout strain displayed a substantial reduction in viability at 800 μM NaNO_2_ (**S6E Fig**). Combining H_2_O_2_ and NaNO_2_ enhanced the sensitivity of the knockout bacteria toward acidified nitrite (where the growth inhibition started at 600 μM concentration) (**S6F Fig**). To ensure OmpA-dependent protection of wild-type *Salmonella* against the *in vitro* nitrosative damage, we performed a death kinetics experiment with the wild-type and knockout bacteria in the presence of 800 μM acidified nitrite (**Fig 5A**). Consistent with our previous observations, the knockout strain began to show a significant growth defect from 6 h post-inoculation. We then performed a nitrite uptake assay of *Salmonella* in MOPS-NaOH buffer with 200 μM of nitrite. We noticed a higher nitrite uptake by STM *ΔompA* and the empty vector strain than the wild-type and complemented bacteria (**Fig 5B**). To verify OmpA-dependent redox homeostasis of *Salmonella* in response to *in vitro* nitrosative stress, we exposed the wild-type and knockout strains harboring pQE60-Grx1-roGFP2 plasmid to 800 μM, 1 mM, and 5 mM (**Fig 5C**) concentrations of acidified nitrite for 15, 30, 45 and 60 minutes. The glutaredoxin (Grx1) fused to the redox-sensitive GFP2 can reversibly transfer electrons between the cellular (GSH/GSSG) pool and the thiol groups of roGFP2 at a much faster rate. The ratio of fluorescence intensity of Grx1-roGFP2 at 405 nm and 488 nm demonstrates the redox status of the cytoplasm of bacteria [42,43]. In all the three concentrations of acidified nitrite, we found a time-dependent increase in the 405/ 488 ratio of Grx1-roGFP2 in STM *ΔompA* strain compared to STM (WT), which suggests a strong redox burst in the cytoplasm of *ompA* knockout strain (**Fig 5C**). Collectively, our data show the importance of OmpA in maintaining the cytosolic redox homeostasis in *Salmonella*. Compared to STM (WT), the enhanced uptake of nitrite by STM *ΔompA* suggested increased permeability of the bacterial outer membrane without OmpA. To further validate this observation, the outer membrane depolarization of *Salmonella* growing in acidic F media was tested (during the stationary phase) using a membrane-permeant negatively charged dye, DiBAC_4_ (**Fig 5D**) [44]. When the outer membrane permeability of the bacteria increases, the negative charge density of the bacterial cytosol is diminished by the inflow of cations (depolarization) from the media. This makes the bacterial cytosol accessible to DiBAC_4,_ which binds to the cell membrane proteins and fluoresces. Contrary to the wild-type and the complemented strain, the higher DiBAC_4_ positive population and greater median fluorescence intensity of DiBAC_4_ corresponding to STM *ΔompA* confirmed its enhanced outer membrane permeability (**Fig 5D**). We then used another porin-specific DNA binding fluorescent dye, bisbenzimide **(Fig 5E)** [41]. Once again, we observed that the fluorescence intensity of bisbenzimide was higher for STM *ΔompA* than for the wild-type strain (**Fig 5E**). Wild-type *Salmonella* pre-treated with saponin (a cell perforating detergents) was used as a positive control for this phenotype. Compared to the wild-type bacteria, the greater fluorescence intensity of bisbenzimide for STM *ΔompA* growing in macrophages for 12 h firmly endorsed the result obtained from the *in vitro* experiment (**Fig 5F**). Earlier, we observed a reduction in the expression level of larger outer membrane porins (*ompC*, *ompD*, and *ompF*) in wild-type *Salmonella* growing in LB broth, acidic F media, and RAW264.7 cells. As deleting *ompA* enhances the outer membrane permeability of *Salmonella*, we decided to study the expression of these larger porins in STM *ΔompA*. Compared to the wild-type and complemented strain, an elevated expression of *ompC*, *ompD*, and *ompF* was observed in STM *ΔompA* growing in LB broth, F media, and macrophages (**Fig 5G**). To understand if the increased expression of these larger porins on bacterial outer membrane enhances its porosity, we expressed *ompC*, *ompD*, and *ompF* (cloned in pQE60 plasmids) in wild-type *Salmonella*. We observed that the increased expression of *ompD* and *ompF* enhanced the outer membrane permeability of *Salmonella* growing in F media (**S10A and S10B Fig**). We then overexpressed *ompC*, *ompD*, and *ompF* in wild-type *Salmonella* by adding IPTG. Our data suggested that the over-expression of *ompF* in *Salmonella* causes massive depolarization (61.34 ± 0.31)% of the outer membrane when compared to *ompC* (3 ± 0.07)% and *ompD* (7.71 ± 0.09)% (**S10C and S10D Fig**). Hence, we concluded that in the absence of *ompA*, the expression of larger porins such as *ompC*, *ompD*, and *ompF* increases on the outer membrane of *Salmonella*. However, the elevated expression of *ompF* majorly regulates the outer membrane porosity of STM *ΔompA*.

### OmpC, OmpD, and OmpF deficiency in *Salmonella* does not hamper the stability of SCV

**Fig 6:**
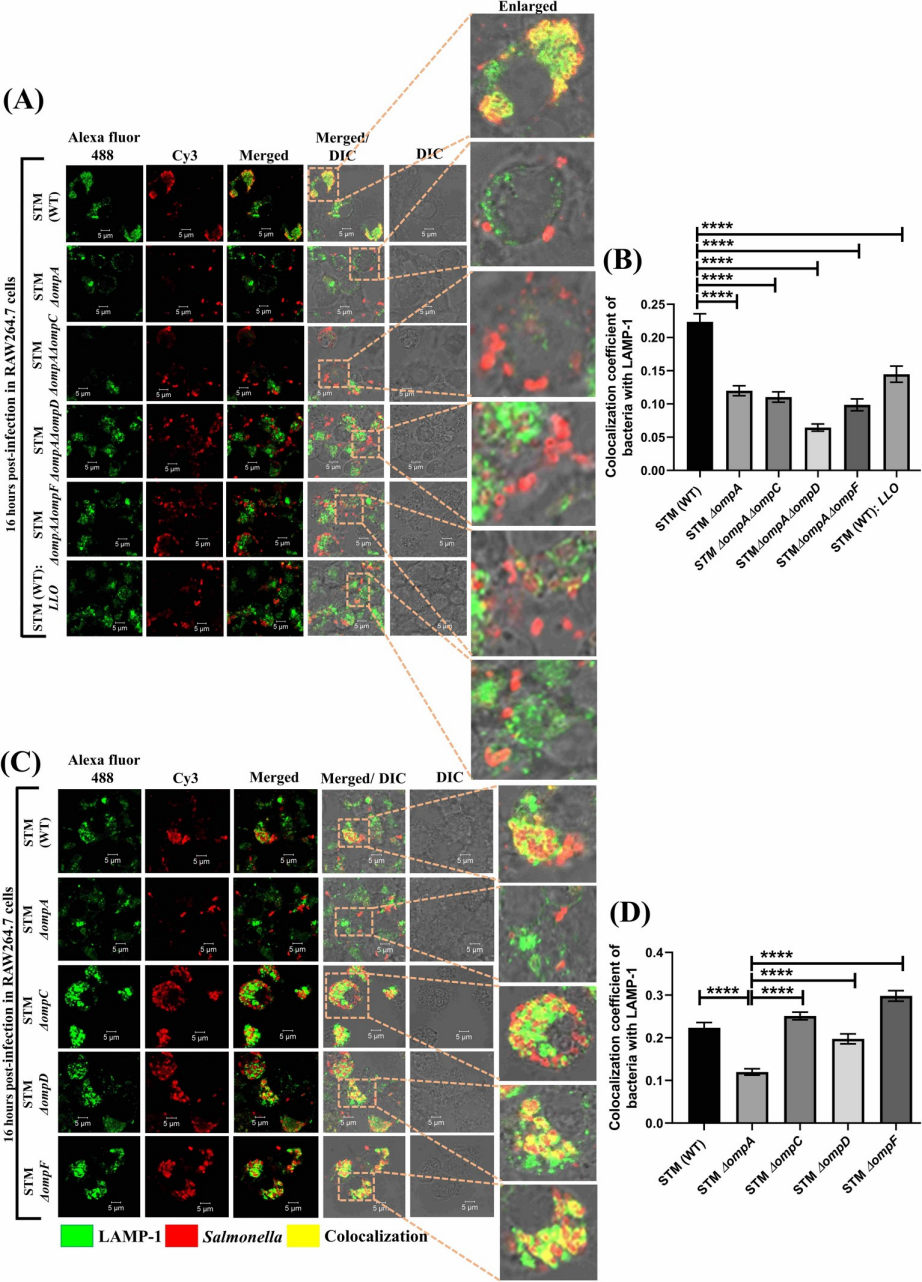
OmpC, OmpD, and OmpF deficiency in *Salmonella* doesn’t hamper the stability of SCV. (A) Representative image of LAMP-1 recruitment on STM (WT), *ΔompA*, *ΔompAΔompC*, *ΔompAΔompD*, *ΔompAΔompF*, & WT: *LLO* (MOI = 20) in RAW264.7 cells. (B) The colocalization coefficient of bacteria with LAMP-1 was represented as a vertical bar graph (n≥50, N = 2). Scale bar = 5μm. (C) RAW264.7 cells were infected with STM (WT), *ΔompA*, *ΔompC*, *ΔompD*, and *ΔompF* (MOI 20) to visualize the intracellular niche of the bacteria. (D) Quantifying the colocalization coefficient of bacteria with LAMP-1 (n≥60, N = 2). Scale bar = 5μm. Data are represented as mean ± SEM. *(P)* ****< 0.0001, (One-way ANOVA).

To study the role of the larger porins in the intracellular virulence of *Salmonella*, we generated double knockout strains (STM *ΔompAΔompC*, *ΔompAΔompD*, and *ΔompAΔompF*) and then investigated their intracellular niche in RAW264.7 cells during the late phase of infection. Consistent with our previous finding, STM *ΔompA* showed poor colocalization with SCV marker LAMP-1 compared to the wild-type bacteria (**Fig 6A and 6B**). The loss of SCV membrane from the surroundings of STM *ΔompAΔompC*, *ΔompAΔompD*, and *ΔompAΔompF*, as demonstrated by the reduced recruitment of LAMP-1 (**Fig 6B**), indicated the cytosolic localization of the double knockout strains in macrophages. To rule out the possibility that lack of OmpC, OmpD and OmpF contributed to the cytosolic localization of STM *ΔompAΔompC*, *ΔompAΔompD*, and *ΔompAΔompF* (**Fig 6A and 6B**), we generated single knockout strains of *Salmonella* lacking *ompC*, *ompD*, and *ompF*. We observed that, similar to the wild-type bacteria, the STM *ΔompC*, *ΔompD*, and *ΔompF* colocalized with LAMP-1 (**Fig 6C and 6D**). Hence, we conclude that the maintenance of the vacuolar life of *Salmonella* depends on OmpA and not on OmpC, OmpD, and OmpF. A decreased recruitment of nitrotyrosine on STM *ΔompC*, *ΔompD*, and *ΔompF* in comparison with STM *ΔompA* in RAW264.7 macrophages confirmed the presence of intact SCV membrane around them (**S11A Fig**). In addition, unlike STM *ΔompA*, the ability of STM *ΔompC*, *ΔompD*, and *ΔompF* to withstand the bactericidal effect of acidified nitrite suggested the dispensability of these larger porins in protecting *Salmonella* from nitrosative stress (**S11B Fig**). In support of this observation, we found that STM *ΔompC*, *ΔompD*, and *ΔompF* are more efficient in restricting the entry of nitrite compared to STM *ΔompA* (**S11C Fig**).

### In the absence of OmpA, outer membrane protein F (OmpF) enhances the susceptibility of *Salmonella* against the nitrosative stress of RAW264.7 cells

**Fig 7:**
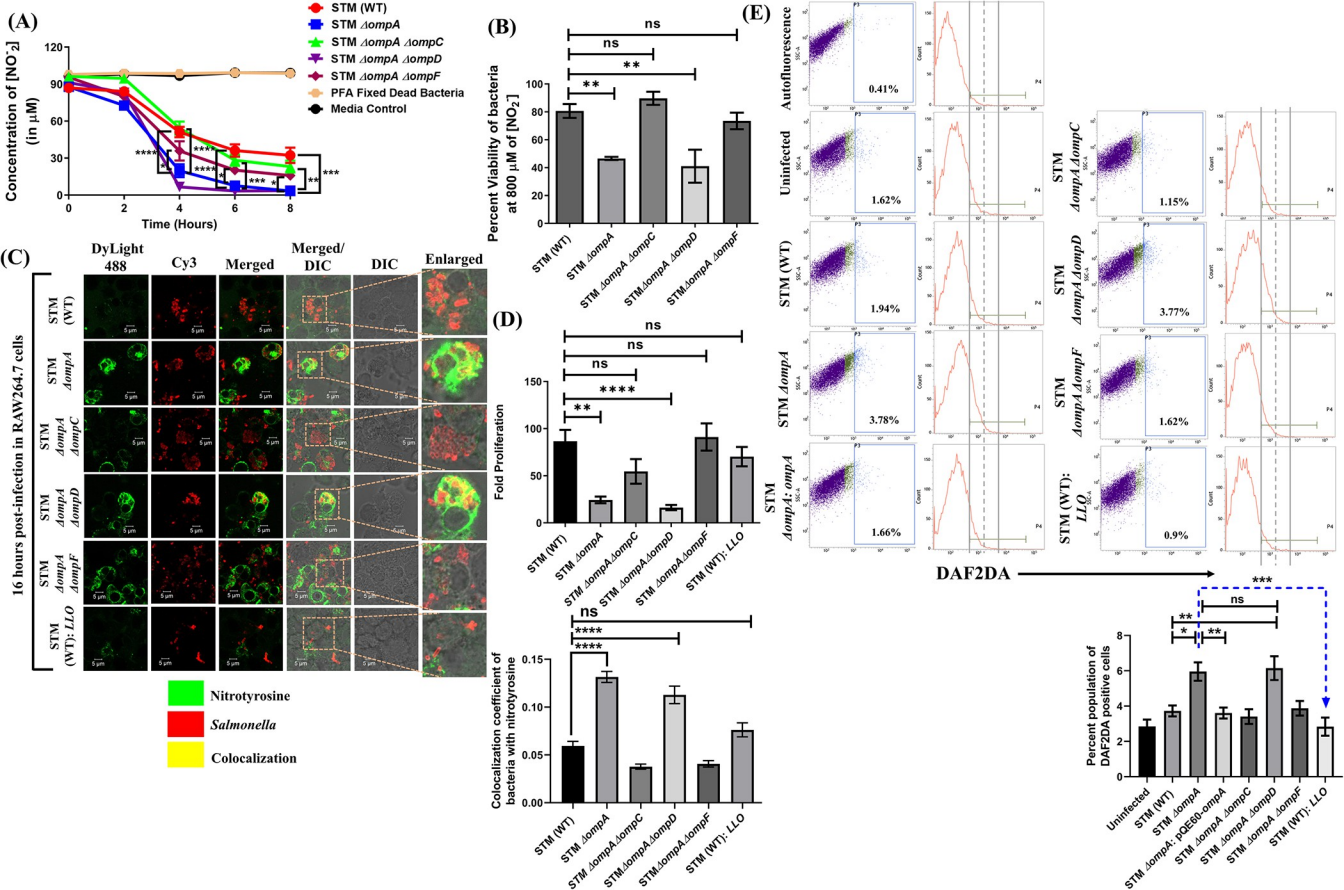
In the absence of OmpA, outer membrane protein F (OmpF) enhances the susceptibility of *Salmonella* against the nitrosative stress of RAW264.7 cells. (A) *In vitro* nitrite uptake assay of STM (WT), *ΔompA*, *ΔompAΔompC*, *ΔompAΔompD*, *ΔompAΔompF*, & PFA fixed dead bacteria (n = 3, N = 6). (B) *In vitro* viability assay of STM (WT), *ΔompA*, *ΔompAΔompC*, *ΔompAΔompD*, & *ΔompAΔompF* in the presence of acidified nitrite at 12 hours post-inoculation using resazurin solution (n = 3, N = 3). (C) Immunofluorescence image of STM (WT), *ΔompA*, *ΔompAΔompC*, *ΔompAΔompD*, *ΔompAΔompF*, & (WT): *LLO* (MOI 20) in RAW264.7 cells to study the recruitment of nitrotyrosine. The colocalization coefficient of bacteria with nitrotyrosine was represented as a vertical bar graph (n≥60, N = 3). Scale bar = 5μm. (D) Calculating the fold proliferation of STM (WT), *ΔompA*, *ΔompAΔompC*, *ΔompAΔompD*, & *ΔompAΔompF*, & (WT): *LLO* respectively (MOI 10) in RAW264.7 cells (n = 3, N = 2). (E) Estimating the level of intracellular NO in RAW 264.7 cells infected with STM (WT), *ΔompA*, *ΔompA*:pQE60-*ompA*, *ΔompAΔompC*, *ΔompAΔompD*, *ΔompAΔompF*, and (WT): *LLO* respectively at MOI 10 using DAF-2DA [5 μM] by flow cytometry. Both dot plots (SSC-A vs. DAF-2 DA) and histograms (Count vs. DAF-2 DA) were represented. The percent population of DAF-2DA positive macrophages has been represented in a bar graph (n≥3, N = 5). Data are represented as mean ± SEM. *(P)* *< 0.05, *(P)* **< 0.005, *(P)* ***< 0.0005, *(P)* ****< 0.0001, ns = non-significant, (Unpaired student’s *t* test in A and one-way ANOVA in B, C, D, E).

To dissect the role of each larger porin in nitrite consumption of *Salmonella* during the absence of OmpA, we performed a nitrite uptake assay using STM *ΔompAΔompC*, *ΔompAΔompD*, and *ΔompAΔompF* (**Fig 7A**). In comparison with the wild-type bacteria, the higher consumption of nitrite by STM *ΔompA* and *ΔompAΔompD* confirmed the involvement of OmpC and OmpF (present in both STM *ΔompA* and *ΔompAΔompD*) in the entry of nitrite (**Fig 7A**). This result was further validated by the reduced *in vitro* viability of STM *ΔompA* and *ΔompAΔompD* in acidified nitrite (800 μM) (**Fig 7B**). While infecting RAW264.7 macrophages, we observed enhanced recruitment of nitrotyrosine on STM *ΔompA* and *ΔompAΔompD* compared to the wild-type, *ΔompAΔompC*, and *ΔompAΔompF* strains of *Salmonella* (**Fig 7C**). To determine the effect of intracellular nitrosative stress on the survival of STM *ΔompAΔompD*, we performed a gentamycin protection assay. It was observed that unlike STM (WT), *ΔompAΔompC*, and *ΔompAΔompF*, which were showing poor colocalization with nitrotyrosine, the intracellular proliferation of STM *ΔompA* and *ΔompAΔompD* was severely compromised in macrophages (**Fig 7D**). Further, our data revealed that only (4.13 ± 0.35)% of macrophages infected with the wild-type *Salmonella* produced NO (**Fig 7E**). This was comparable to the macrophages infected with STM *ΔompA*: pQE60-*ompA* (3.98 ± 0.33)%, *ΔompAΔompC* (3.74 ± 0.46)%, and *ΔompAΔompF* (4.21 ± 0.46)% (**Fig 7E**). On the contrary, the greater percent population of DAF2DA positive macrophages corresponding to STM *ΔompA* (6.43 ± 0.56)% and *ΔompAΔompD* (6.59 ± 0.71)% validated the observation obtained from confocal imaging (**Fig 7E**). Taken together, we conclude that in the absence of OmpA, the elevated expression of OmpC and OmpF on the bacterial outer membrane helped in the entry of RNI into the bacterial cytoplasm and made the bacteria susceptible to the intracellular nitrosative stress. To validate this observation, we incubated the wild-type *Salmonella*, expressing *ompA*, *ompC*, *ompD*, and *ompF* in acidified nitrite for 12 hours and quantified their viability by propidium iodide staining (**S12A–S12C Fig**) and resazurin assay (**S12D–S12F Fig**). Earlier, we have shown that over-expression of *ompD* and *ompF* enhances the permeability of the outer membrane of wild-type *Salmonella*. Consistent with this observation, the flowcytometric data showed that acidic PBS induced a significant amount of death in wild-type *Salmonella* expressing *ompD* (11.81 ± 1.08)% and *ompF* (8.75 ± 0.5)% (**S12A and S12B Fig**). However, no significant reduction in their viability was observed in the resazurin assay (**S12D and S12F Fig**). When the overexpression strains were incubated in acidified nitrite, it was found that STM (WT): *ompF* (20.54 ± 0.5)% survived less compared to STM (WT): *ompD* (15.71 ± 0.3)% (**S12A and S12C Fig**). This observation was further supported by the resazurin assay showing compromised viability of STM (WT): *ompF* (60.17 ± 1.45)% compared to STM (WT): *ompD* (100.8 ± 2.98)% in response to acidified nitrite (**S12E and S12F Fig**), suggesting that the upregulated expression of OmpF in STM *ΔompA* increased its susceptibility towards *in vitro* and *in vivo* nitrosative stress.

## Discussion

Bacterial pathogens can restrict the entry of toxic molecules such as antibiotics, bile salts, and cationic antimicrobial peptides by changing their outer membrane permeability or augmenting omptins [45–47]. The alteration in the outer membrane permeability of Gram-negative pathogen is strictly regulated by the core oligosaccharide composition of lipopolysaccharide and differential expression of outer membrane porins [48–50]. OmpA is one of the most abundant outer membrane porins of *Salmonella* Typhimurium. It tightly anchors the outer membrane of the bacteria to the peptidoglycan layer of the cell wall. Hence, the deletion of OmpA aggravates the biogenesis of outer membrane vesicles (OMV) [8,51]. Apart from OmpA, other major porins present on the outer membrane of *Salmonella* help in transporting charged molecules [9,52]. However, very few studies have addressed the correlation between outer membrane porins and *Salmonella* pathogenesis. A previous study from our group reported that OmpA deficient *Salmonella* could not reach the mouse brain [53]. The alternative sigma factor of *Salmonella* regulates the expression of OmpA within macrophages [54]. In the current study, we focused on deciphering the role of OmpA in *Salmonella* pathogenesis.

Unlike other significant porins, *Salmonella* OmpA has a small pore size and a unique periplasmic domain (**S1A–S1D Fig**), which can act as a gate to restrict the entry of many toxic molecules [10]. The increased expression of *ompA* in the wild-type *Salmonella* growing in macrophages proved the requirement of this porin for its intracellular survival. Besides their structural role, porins can interact with host immune cells. An eight-stranded β-barrel outer membrane porin (*ompW*) of *Escherichia coli* helps the bacteria evade phagocytosis and confers resistance against alternative complement activation pathway [6,7]. OmpA of *E*. *coli* K1 augments complement resistance by recruiting C4BP and aggravates the intracellular survival of the bacteria in murine and human macrophages [55,56]. In our study, the OmpA deficient strain of *Salmonella* has shown increased phagocytosis and severely attenuated intracellular proliferation in macrophages. Surprisingly, STM *ΔompA* was invasion-deficient and hyper-proliferative in the epithelial cells. The successful intracellular survival of *Salmonella* depends upon its life within the SCV. *Salmonella* recruits a plethora of SPI-encoded virulent factors and host proteins to maintain the stability of SCV. *SifA* mutant of *Salmonella* disrupts the SCV and comes into the cytosol of the host cells [57]. Introducing point mutations in Rab5 and Rab7 can also release the wild-type bacteria into the cytosol of epithelial cells [58]. Interestingly, our study reported the release of STM *ΔompA* in the cytosol of macrophages and epithelial cells. The disruption of SCV can induce autophagy in the host cell [59–62]. The increased colocalization of STM *ΔompA* with syntaxin 17 and LC3B in macrophages proved the disruption of SCV in the absence of OmpA [63]. We found that STM *ΔompA* growing in LB broth is deficient in expressing SPI-2 effector genes such as *sseC*, *sseD*, *sifA*, and *ssaV*. The reduced expression of *sifA* in STM *ΔompA* could be the reason behind its cytosolic localization in macrophages and epithelial cells. The acidification of the bacteria inside the SCV is a prerequisite for synthesizing and secreting SPI-2 encoded effector proteins [22]. Multiple studies reported that Gram-negative pathogens use outer membrane porins for pH sensing and homeostasis [64–67]. Consistent with this observation, we found that the reduced expression of SPI-2 effectors in STM *ΔompA* was not restored in SPI-2-inducing acidic F media, suggesting impaired pH homeostasis of the bacteria in the absence of OmpA. The SCV membrane serves as a protective barrier around wild-type bacteria. Once the integrity of the SCV membrane is breached, the bacteria are exposed to ROS and RNI present in the cytosol of the host cells [33]. Bonocompain. G *et al*. reported that *Chlamydia trachomatis* infection in HeLa cells transiently induces ROS [68]. The epithelial cells (HeLa) cannot challenge wild-type *Salmonella* with ROS during infection as efficiently as the macrophages [33]. However, stimulation of colonic epithelial cells with bacterial infection can induce RNI [69]. In our study, we observed a lack of difference in the level of intracellular NO in Caco-2 cells infected with the wild-type and *ompA* mutant bacteria, explaining the reason for excessive growth of STM *ΔompA* in epithelial cells. On the contrary, STM *ΔompA* induced a higher NO response in macrophages, which reduced its proliferation. Wild-type *Salmonella* uses SPI- 2 encoded virulent factor SpiC to activate the suppressor of cytokine signaling 3 (SOCS-3), which inhibits IFN-γ signaling and thus eventually represses the activity of iNOS [36,37]. STM *ΔompA*, deficient in producing SpiC in *in vitro* and *in vivo* growth conditions, couldn’t suppress iNOS activity in the macrophages and showed a higher NO response. Continuing with this observation, STM *ΔompA*, staying in the cytosol of macrophages, showed greater colocalization with nitrotyrosine compared to the wild-type bacteria protected inside the SCV. The intracellular population of *Listeria monocytogenes* utilizes LLO to degrade the phagosomal membrane for escaping lysosomal fusion [34,70]. A decreased recruitment of nitrotyrosine on the cytosolic population of STM (WT): *LLO* and their better survival compared to STM *ΔompA* and *ΔompA*: *LLO* showed the role of OmpA in defending the cytosolic population of *Salmonella* from the harmful effect of RNI. In addition, the enhanced recruitment of nitrotyrosine and compromised intracellular survival of STM *ΔompAsifA* and *ΔompAssaV* compared to STM *ΔsifA*, and *ΔssaV* showed a SPI-2 independent additional role of OmpA in protecting the bacteria from the damage caused by nitrosative stress.

**Fig 8:**
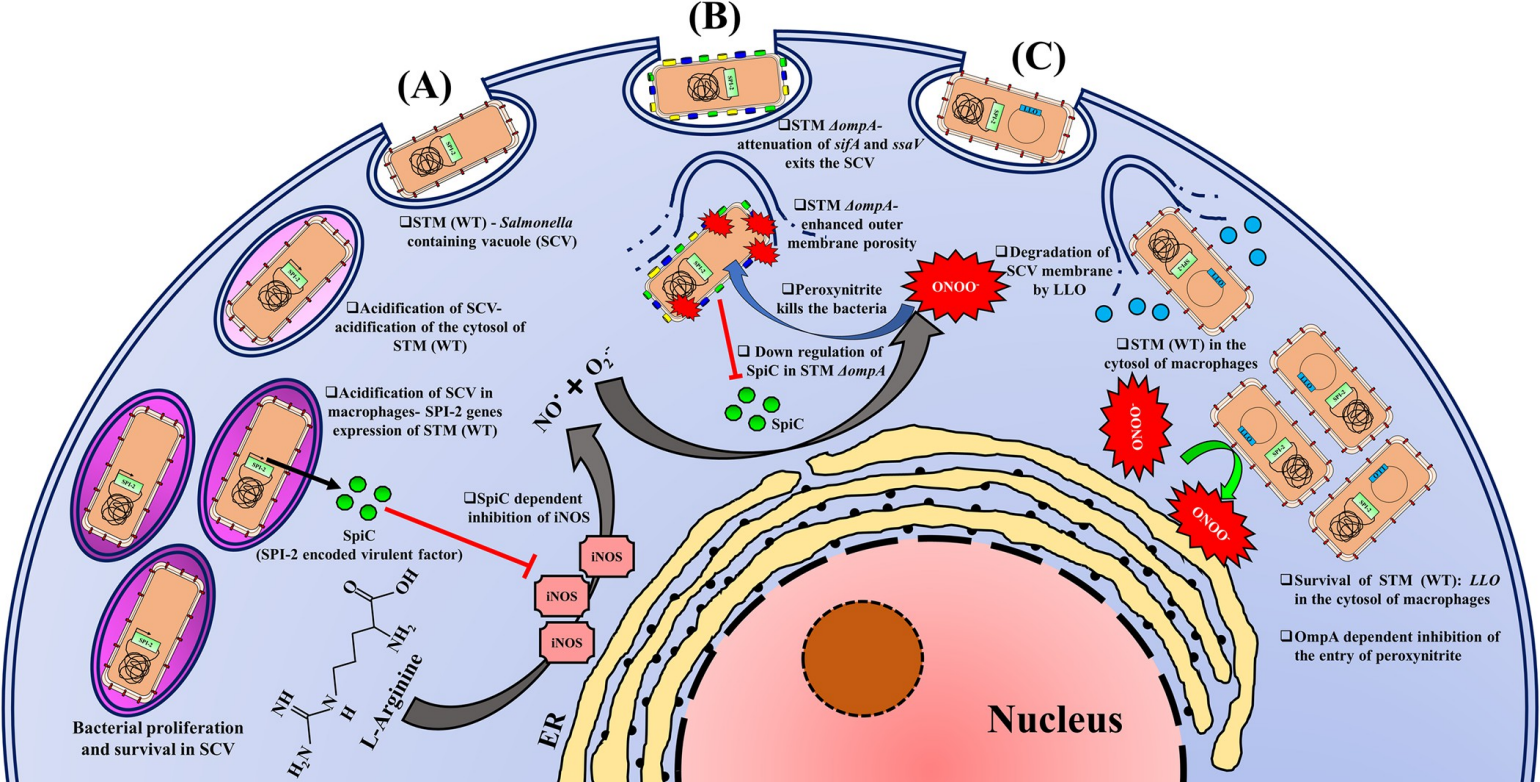
The hypothetical working model of intracellular survival of STM (WT), STM *ΔompA*, and STM (WT): *LLO*. The hypothetical model depicts the fate of (A) STM (WT), (B) STM *ΔompA*, and (C) STM (WT): *LLO* inside the murine macrophages. (A) STM (WT) staying inside the acidic SCV can proliferate efficiently by suppressing the activity of iNOS by SPI-2 encoded virulent factors SpiC. The acidification of the cytosol of wild-type bacteria due to the acidic pH of SCV triggers the expression of SPI-2 genes. (B) STM *ΔompA*, deficient in expressing SPI-2 effector genes *sifA* and *ssaV*, comes into the cytosol of macrophages after disrupting the SCV. It is unable to produce SpiC and cannot suppress the activity of iNOS. The enhanced outer membrane permeability of the cytosolic population of STM *ΔompA* due to the upregulation of *ompF* makes them vulnerable to RNI. (C) STM (WT): *LLO* exits the SCV by expressing LLO. Unlike STM (WT), the cytosolic niche of STM (WT): *LLO* cannot produce SpiC. It can protect itself from RNI by reducing its outer membrane permeability by expressing *ompA* and surviving efficiently in the cytosol.

Our data further revealed that STM *ΔompA* could not induce ROS while infecting macrophages. NADPH phagocytic oxidase is the key enzyme that produces super oxides ions in macrophages [71]. Wild-type *Salmonella* can impair the recruitment of NADPH oxidase on the surface of the SCV membrane in a SPI-2 encoded T3SS-dependent manner [72]. The restriction of the recruitment of NADPH oxidase on the damaged SCV membrane of STM *ΔompA* is the probable reason behind the decreased oxidative stress response inside the macrophages. On the contrary, the ability of iNOS to maintain its uninterrupted catalytic activity while being recruited on the cortical actin of macrophages might continue the biogenesis of RNI during STM *ΔompA* infection [73]. The reduced bacterial burden in the organs and better survival of the mice infected with STM *ΔompA* endorsed an important role of OmpA in the *in vivo* pathogenesis of *Salmonella*. The reversal of the attenuated proliferation of STM *ΔompA* under the inhibition of *in vivo* iNOS activity suggested OmpA-dependent protection of wild-type bacteria against the *in vivo* nitrosative stress. Acidified nitrite generating a wide range of reactive nitrogen intermediates (RNI) causes irreparable damage to bacteria and fungi [40,74]. The enhanced sensitivity of STM *ΔompA* to acidified nitrite and the combination of acidified nitrite with peroxide indicated an increased entry of nitrite due to severe damage in their outer membrane [75]. Earlier, Choi and Lee *et al*. observed that the OmpA mutant of *E*.*coli* possessed a leaky outer membrane [50]. Consistent with this observation, our study revealed that the OmpA of *Salmonella* Typhimurium helps in maintaining the integrity of the outer membrane. A significant increase in the expression of the larger porins (*ompC*, *ompD*, and *ompF*) in STM *ΔompA* resulted in enhanced outer membrane permeability, which in turn made the bacteria susceptible to the nitrites. This conclusion is corroborated by the increased outer membrane permeability of wild-type *Salmonella* overexpressing *ompD* and *ompF*. We observed that STM *ΔompAΔompD*, possessing intact OmpC and OmpF, showed enhanced nitrite consumption and higher sensitivity to *in vitro* nitrosative stress. Consistent with this, greater recruitment of nitrotyrosine on the cytosolic population of STM *ΔompAΔompD* reduced its proliferation in the macrophages. Unlike STM *ΔompAΔompD*, the inability of STM *ΔompAΔompC* and *ΔompAΔompF* to induce NO response while infecting the macrophages further confirmed the observation. In line with these findings, we observed that the over-expression of *ompF* in wild-type *Salmonella* remarkably increased its susceptibility to acidified nitrites. This further explains the reason behind the enhanced recruitment of nitrotyrosine on STM *ΔompA* compared to the cytosolic population of STM (WT): *LLO* in macrophages. STM (WT): *LLO* has intact OmpA, which maintains the integrity of its outer membrane and inhibits the entry of peroxynitrite **(Fig 8)**. The loss of stability in the outer membrane of STM *ΔompA* due to the upregulation of *ompF* made the bacteria accessible to peroxynitrites. These data collectively suggest the pivotal role of OmpF in the entry of nitrite in *ompA* deficient *Salmonella* by increasing the permeability of the outer membrane. In this context, we must mention that STM *ΔompAΔompC* is also expected to express OmpD and OmpF. The better survival of STM *ΔompAΔompC* compared to STM *ΔompAΔompD* in response to nitrosative stress will be answered in the future.

To summarize, our study proposed a novel OmpA-dependent mechanism employed by *Salmonella* to protect itself from the damage caused by the nitrosative stress of macrophages. Deleting *ompA* from *Salmonella* reduced the expression of SPI-2 effector genes and released the mutant bacteria into the cytosol from SCV. The intracellular proliferation of STM *ΔompA* was severely attenuated in macrophages due to increased NO response. We have provided conclusive evidence that OmpA maintains the stability of the outer membrane of *Salmonella*. In the absence of OmpA, the enhanced expression of OmpF increased the porosity of the outer membrane and made the bacteria susceptible to *in vitro* and *in vivo* nitrosative stress.

## Materials and methods

### Ethics statement

The animal experiments were approved by the Institutional Animal Ethics Committee (IAEC) at Indian Institute of Science, Bangalore, India (Registration No: 48/1999/CPCSEA) and the guidelines provided by the CPCSEA was strictly flowed. The Committee for the Purpose of Control and Supervision of Experiments on Animals (CPCSEA) was established under Chapter 4, Section 15(1) of the Prevention of Cruelty to Animals Act 1960. Ethical clearance number for this study is CAF/Ethics/670/2019.

### Bacterial strains, media, and culture conditions

The wild-type *Salmonella enterica* serovar Typhimurium strain 14028S used in this study was a generous gift from Professor Michael Hensel, Max Von Pettenkofer-Institute for Hygiene und Medizinische Mikrobiologie, Germany. The bacterial strains were revived from glycerol stock (stored at -80°C) and plated on LB agar with or without appropriate antibiotics. The LB broth cultures of wild-type, knockout, and complemented strains were grown in a shaker incubator at 37°C (180 rpm). The strains expressing pKD46 and harboring pQE60-Grx1-roGFP2 (under IPTG treatment) were grown overnight in a shaking incubator at 30°C. For growth curve experiments and *in vitro* RNA extraction studies, a single colony was inoculated in 5mL LB broth and grown overnight at 37°C. The stationary phase bacteria were sub-cultured at a 1: 100 ratio in freshly prepared LB or minimal F media (acidic) and kept in a 37°C shaker incubator. At different time intervals, aliquots were taken for RNA isolation, serial dilution, plating, and [OD]_600nm_ measurement by TECAN 96 well microplate reader. This table shows the complete list of strains, plasmids, and antibiotics **(Table 1)**.

**Table 1:** Strains and plasmids used in this study.

Strains/ plasmids	Characteristics	Source/ references
*Salmonella enterica* serovar Typhimurium ATCC strain14028S	Wild type (WT)	Gifted by Prof. M. Hensel
*S*. Typhimurium *ΔompA*	Kan^R^	This study
*ΔompA*: pQE60-*ompA*	Kan^R^, Amp^R^	This study
*ΔompA*: pQE60	Kan^R^, Amp^R^	This study
*S*. Typhimurium *ΔompC*	Chl^R^	This study
*S*. Typhimurium *ΔompD*	Chl^R^	This study
*S*. Typhimurium *ΔompD*	Kan^R^	This study
*S*. Typhimurium *ΔompF*	Chl^R^	This study
*S*. Typhimurium *ΔsifA*	Chl^R^	This study
*S*. Typhimurium *ΔssaV*	Chl^R^	This study
*S*. Typhimurium *ΔompAΔompC*	Kan^R^, Chl^R^	This study
*S*. Typhimurium *ΔompAΔompD*	Kan^R^, Chl^R^	This study
*S*. Typhimurium *ΔompAΔompF*	Kan^R^, Chl^R^	This study
*S*. Typhimurium *ΔompAΔsifA*	Kan^R^, Chl^R^	This study
*S*. Typhimurium *ΔompAΔssaV*	Kan^R^, Chl^R^	This study
pKD4	Plasmid with FRT-flanked kanamycin resistance gene	[14]
pKD46	Plasmid expressing λ red recombinase, Amp^R^	[14]
pQE60 vector	Low copy number plasmid, Amp^R^	Laboratory stock
pFV- mCherry (RFP)	Amp^R^	Laboratory stock
pFV: GFP	Amp^R^	Laboratory stock
STM (WT): *LLO*	Amp^R^	Laboratory stock
STM *ΔompA*: *LLO*	Amp^R^	This study
*S*. Typhimurium wild type: pHG86 *spiC-LacZ*	Amp^R^	This study
*S*. Typhimurium *ΔompA*: pHG86 *spiC-LacZ*	Amp^R^	This study
*S*. Typhimurium wild-type: pQE60-*ompA*	Amp^R^	This study
*S*. Typhimurium wild-type: pQE60-*ompC*	Amp^R^	This study
*S*. Typhimurium wild-type: pQE60-*ompD*	Amp^R^	This study
*S*. Typhimurium wild-type: pQE60-*ompF*	Amp^R^	This study
*S*. Typhimurium wild-type: pQE60	Amp^R^	This study
pHG86 *spiC-LacZ*	Amp^R^	Laboratory stock
pQE60-Grx1-roGFP2	Amp^R^	Gifted by Dr. Amit Singh, CIDR, IISc

### Eukaryotic cell lines and growth conditions

The RAW264.7, HeLa, and Caco-2 cells were maintained in Dulbecco’s Modified Eagle’s Media (Sigma-Aldrich) supplemented with 10% FCS (Fetal calf serum, Gibco) at 37°C temperature in the presence of 5% CO_2_. U937 cells were maintained in Roswell Park Memorial Institute 1640 media (Sigma-Aldrich) supplemented with 10% FCS. DMEM with 1% non-essential amino acid solution (Sigma-Aldrich) was used to polarize the Caco-2 cells. U937 cells were incubated with Phorbol Myristate Acetate (Sigma-Aldrich) (20 ng/ mL) for activation (for 24 hours). This media was replaced with normal RPMI supplemented with 10% FCS and further incubated the cells for 24 hours before starting the experiments.

### Construction of knockout strains of *Salmonella*

The knockout strains of *Salmonella* were made using one step chromosomal gene inactivation method demonstrated by Datsenko and Wanner [14]. Briefly, STM (WT) was transformed with pKD46 plasmid, which has a ‘lambda red recombinase system’ under arabinose inducible promoter. The transformed cells were grown in LB broth with ampicillin (50 μg/mL) and 50 mM arabinose at 30°C until the [OD]_600nm_ reached 0.35 to 0.4. Electrocompetent STM (WT): pKD46 cells were prepared by washing the bacterial cell pellet with double autoclaved chilled Milli Q water and 10% (v/v) glycerol. Kanamycin (Kan^R^, 1.6 kb) and chloramphenicol (Chl^R^, 1.1 kb) resistant gene cassettes were amplified from pKD4 and pKD3 plasmids, respectively, using knockout primers **(Table 2)**. The amplified Kan^R^ and Chl^R^ gene cassettes were electroporated into STM (WT): pKD46. The transformed cells were plated on LB agar with appropriate antibiotics to select the knockout colonies. The knockout colonies were confirmed by confirmatory and kanamycin/ chloramphenicol internal primers **(Table 2)**.

**Table 2:** Primer sequences (5’ to 3’).

Genes	Sequence (5’-3’)
*ompA* knockout forward-	TCGTTGGAGATATTCATGGCGTATTTTGGATGATAACGAGCATATGAATATCCTCCTTAG
*ompA* knockout reverse-	AAGAAGTAACGCTGAAAGGCGTTGTCATCCAGACCAGAGCGTGTAGGCTGGAGCTGCTTC
*ompC* knockout forward-	ATAACTGTAACATCTTAAAAGTTTTAGTATCATATTCGTGGTGTAGGCTGGAGCTGCTTC
*ompC* knockout reverse-	TATCAAAACGTCGTATTTGTACGCCGGAATAAGGCATGATGGGAATTAGCCATGGTCC
*ompD* knockout forward-	TTATTAAAATGAAACTTAAGTTAGTGGCAGTGGCAGTGTTTAAATGGCGCGCCTTACG
*ompD* knockout reverse-	CAAAATTAGAACTGGTAGTTCAGACCAACAGCAACGATGTGGAAGATCACTTCGCAGAA
*ompF* knockout forward-	ATTGACGGAATTTATTGACGGCAGTGGCAGGTGTCATAGTGTAGGCTGGAGCTGCTTC
*ompF* knockout reverse-	TACAAAATGCCAACCGTTAGCGCTAAAAAGCCCGCCTGTTATGGGAATTAGCCATGGTCC
*sifA* knockout forward-	GGGTCGATTTAATCAATTATGTAGTCATTTTTACTCCAGGTGTAGGCTGGAGCTGCTTC
*sifA* knockout reverse-	AAACCCTGAACGTGACGTCTGAGAAAGCGTCGTCTGATATGGGAATTAGCCATGGTCC
*ssaV* knockout forward-	GGTTACGATTACATCATCGACAAATAAAATTTCTGGAGTCGTGTAGGCTGGAGCTGCTTC
*ssaV* knockout reverse-	ATCGGGGGGCGGATATTTCAGCCTCAGACGTTGCATCAATGGGAATTAGCCATGGTCC
*ompA* cloning forward-	CATGCCATGGATGAAAAAGACAGCTATCGC
*ompA* cloning reverse-	CCCAAGCTTTTGTCATCCAGACCAGAG
*ompC* cloning forward-	CGCGGATCCATGAAAGTTAAAGTACTGTCC
*ompC* cloning reverse-	CCCAAGCTTGCTGATTAGAACTGGTAAACC
*ompD* cloning forward-	CGCGGATCCATGAAACTTAAGTTAGTGGC
*ompD* cloning reverse-	CCCAAGCTTCTACAACAAAATTAGAACTGG
*ompF* cloning forward-	CGCGGATCCATGATGAAGCGCAAAATCC
*ompF* cloning reverse-	CCCAAGCTTTCAGAACTGGTAAGTAATACC
*ompA* confirmatory forward-	CGGTAGAGTAACTATTGAG
*ompA* confirmatory reverse-	TTACAGGCGTTATTAGGC
*ompA* expression forward-	ATCCAATCACTGACGATCTG
*ompA* expression reverse-	GCATCACCGATGTTGTTAGT
*ompC* confirmatory forward-	GGTAAACAGACATTCAGA
*ompC* confirmatory reverse-	AGTCATTTTCATCGCTGTT
*ompD* confirmatory forward-	GAACTTATGCCACTCCGTCATT
*ompD* confirmatory reverse	CAGCATTTCGACGTCAACGGTA
*ompF* confirmatory forward-	GTCAGACACATAAAGACACC
*ompF* confirmatory reverse-	CGAGGTTCCATTATAGTTACAG
*sifA* confirmatory forward-	TATTACATCCGATGCGCCCG
*sifA* confirmatory reverse-	CTCAGTAGGCAAACAGGAAGT
*ssaV* confirmatory forward-	TTGTTCTCCACCTCTTTCCA
*ssaV* confirmatory reverse-	GTTGCGCTGACATCCTGAAT
Kanamycin^R^ internal forward-	CGGTGCCCTGAATGAACTGC
Kanamycin^R^ internal reverse-	CGGCCACAGTCGATGAATCC
Chloramphenicol^R^ internal forward-	ACAAACGGCATGATGAACCT
Chloramphenicol^R^ internal reverse-	GCTCTGGAGTGAATACCACG
*spiC* expression forward-	ACCTAAGCCTTGTCTTGCCT
*spiC* expression reverse-	CCATCCGCTGTGAGCTGTAT
*sseC* expression forward-	TTTGGCGAGGAAGTGGTTGA
*sseC* expression reverse-	AGCCATTTCACGTTCAAGCG
*sseD* expression forward-	TGTTGTCGGGTGTACTGACG
*sseD* expression reverse-	ACGGCTTGACCCGCTATAAG
*sifA* expression forward-	CCACACGAGAGCGGCTTACA
*sifA* expression reverse-	GCCGTCATTTGTGGATGCGA
*ssaV* expression forward-	CGCCGCAAAAAGTCTGTGGT
*ssaV* expression reverse-	GGGACGCCGGTATCCTCAAA
*16srRNA* forward-	GAGCGCAACCCTTATCCTTTG
*16srRNA* reverse-	CACTTTATGAGGTCCGCTTGCT

### Construction of complemented and overexpression strains of *Salmonella*

The *ompA*, *ompC*, *ompD*, and *ompF* genes were amplified by colony PCR with their respective cloning primers **(Table 2)**. The amplified PCR products and empty pQE60 vector were subjected to restriction digestion by specific restriction enzymes such as NcoI (NEB) and HindIII (NEB) for *ompA*, BamHI (NEB), and HindIII (NEB) for *ompC*, *ompD*, and *ompF* in the CutSmart buffer (NEB) at 37°C for 2–3 h. Double digested insert and vector were subjected to ligation by a T_4_ DNA ligase in 10X ligation buffer (NEB) overnight at 16°C. The ligated products and the empty vector were transformed into the bacteria to generate complemented, over-expression, and empty vector strains. Complementation and over-expression were initially confirmed by restriction digestion of recombinant plasmid. The expression level of *ompA* in the knockout, complemented, and empty vector strains were further confirmed by RT-qPCR using *ompA* expression primer **(Table 2)**.

### Construction of double knockout strains of *Salmonella*

The double knockout strains of *Salmonella* were prepared by slightly modifying the one-step chromosomal gene inactivation strategy demonstrated by Datsenko and Wanner [14]. Briefly, STM *ΔompA* was transformed with pKD46 plasmid. The transformed cells were grown in LB broth with ampicillin (50 μg/mL) and 50 mM arabinose at 30°C until [OD]_600nm_ reached 0.35 to 0.4. Chloramphenicol resistant gene cassette (Chl^R^, 1.1 kb), amplified from pKD3 plasmid using knockout primers **(Table 2)**, was electroporated into STM *ΔompA*: pKD46. The transformed cells were plated on LB agar with kanamycin (50 μg/mL) and chloramphenicol (25 μg/mL) for selecting double knockout strains. The knockout colonies were confirmed by confirmatory and expression primers **(Table 2)**.

### RNA isolation and RT-qPCR

The bacterial cell pellets were lysed with TRIzol reagent (RNAiso Plus) and stored at -80°C overnight. The lysed supernatants were further subjected to chloroform extraction followed by precipitation by adding an equal volume of isopropanol. The pellet was washed with 70% RNA-grade ethanol, air-dried, and suspended in 20 μL of DEPC treated water. RNA concentration was measured in nano-drop and analyzed on 1.5% agarose gel to assess the quality. To make cDNA, 3 μg of RNA sample was subjected to DNase treatment in the presence of DNase buffer (Thermo Fischer Scientific) at 37°C for 2 h. The reaction was stopped by adding 5mM Na_2_EDTA (Thermo Fischer Scientific), followed by heating at 65°C for 10 min. The cDNA was prepared using the PrimeScript RT reagent Kit provided by TaKaRa (Cat# RR037A). Quantitative real-time PCR was done using SYBR/ TB Green RT-qPCR kit in Bio-Rad real-time PCR detection system. The expression level of target genes was measured using specific RT/ expression primers (**Table 2**). 16S rRNA was used to normalize the expression levels of the target genes.

### Infection protocol

0.15 to 0.2 million RAW264.7 and PMA activated U937 monocyte-derived macrophages were infected with stationary phase culture of wild-type, mutant, and complement bacterial strains growing overnight in LB broth (OD_600_ 0.3). The multiplicity of infection (MOI) 10 was used for most of the infection studies. MOI of 50 was used to study the phagocytosis of *Salmonella* in the presence of mouse complement sera. We used MOI of 20 for the confocal study. The Caco-2 and HeLa cells (0.15 to 0.2 million) were infected with mid-log phase culture of required bacterial strains growing in LB (OD_600_ 0.3). MOI of 50 was used to study the bacterial adhesion on RAW264.7 and HeLa cells. The infected cells were treated with a 100 μg/ mL concentration of gentamycin for an hour to kill the extracellular bacteria. After 1 hour, the cells were supplemented with a reduced concentration of gentamycin (25 μg/ mL) and kept until lysis.

### Percent phagocytosis calculation/ invasion assay

RAW264.7 and activated U937 cells were infected with STM (WT), *ΔompA*, *ΔompA*: pQE60-*ompA*, and *ΔompA*: pQE60 at MOI of 10. MOI 50 was used for the bacteria treated with 10% mouse complement sera to study the complement-mediated phagocytosis of macrophages. Caco-2 and HeLa cells were infected with the mid-log phase culture of all four bacterial strains at MOI 10. The infected cells were centrifuged at 800 rpm for 5 min, followed by incubating the infected cells at 37°C in the presence of 5% CO_2_ for 25 min. Next, the cells were washed with PBS to remove unattached bacteria and subjected to 100 μg/ mL and 25 μg/ mL concentration of gentamycin treatment for 1 h each. 2 h post-infection, the cells were lysed with 0.1% triton-X-100. The lysate was plated on *Salmonella- Shigella* agar, and the corresponding CFUs were enumerated. Percent phagocytosis (for macrophage cells)/ percent invasion (for epithelial cells) was determined using the following formula-

Percentphagocytosis/percentinvasion=[CFUat2h]/[CFUofpre‐inoculum]*100

### Adhesion assay

The protocol of adhesion assay has been followed as described earlier [17]. Briefly, infected RAW264.7 and HeLa cells were incubated at 37°C temperature in the presence of 5% CO_2_ for 15 and 25 minutes, respectively. After incubation, the cells were washed with sterile PBS and fixed with 3.5% PFA. To visualize the externally attached bacteria, the cells were primarily treated with rabbit-raised anti-*Salmonella* antibody (dilution 1: 100, duration 6 to 8 hours at 4°C temperature), which was followed by anti-rabbit Alexa fluor 488 secondary antibody (dilution 1: 200, duration 1 hour at room temperature), dissolved in 2.5% BSA solution without saponin. Images were obtained by confocal laser scanning microscopy (Zeiss LSM 710) using a 63X oil immersion objective lens. The number of bacteria adhering per cell was calculated by dividing the total number of bacteria attached by the total number of host cells in a single microscopic field. The counting and analysis were done with the help of ZEN Black 2009 software provided by Zeiss.

### Intracellular proliferation assay/ Gentamycin protection assay

The protocol of intracellular proliferation assay has been followed, as demonstrated earlier [53]. Briefly, the seeded RAW264.7, U937, Caco-2, and HeLa cells were infected with STM (WT), *ΔompA*, *ΔompA*: pQE60-*ompA*, and *ΔompA*:pQE60 at MOI 10. After centrifuging the cells at 800 rpm for 5 minutes, the infected cells were incubated at 37°C in the presence of 5% CO_2_ for 25 minutes. Next, the cells were washed thrice with PBS to remove all the unattached extracellular bacteria and subjected to 100 μg/ mL concentrations of gentamycin treatment for 1 hour. This was followed by incubating the cells with 25 μg/ mL concentrations of gentamycin till the lysis. The cells were lysed with 0.1% triton-X-100 at 2 hours and 16 hours post-infection. The lysates were plated on *Salmonella- Shigella* Agar, and the corresponding CFU was determined at 2 and 16 hours. The intracellular proliferation of bacteria (Fold proliferation) was determined using a simple formula-

Foldproliferation=[CFUat16hours]/[CFUat2hours]

In some sets of experiments, the fold proliferation of STM (WT) and *ΔompA* in the macrophages (RAW 264.7) was measured in the presence of 1400W dihydrochloride [10μM] and mouse IFN-γ [100U/ mL]. 1400W and IFN-γ were added to the infected cells with 25 μg/ mL of gentamycin solution. For plating, two consecutive dilutions were made from each technical replicate at 2 and 16 hours. The CFU obtained from each dilution was used to calculate the fold proliferation.

### Chloroquine resistance assay

The chloroquine resistance assay was performed to estimate the number of intracellular bacteria localized in the cytosol using a modified protocol [76–78]. Briefly, the RAW264.7 and Caco-2 cells were infected with STM (WT), *ΔompA*, and *ΔompA*: pQE60-*ompA* at MOI 10. Infected cells were treated with gentamycin, as described earlier. The cells were supplemented with 50 μg/ mL of chloroquine two hours before lysis (14 hours post-infection) and lysed with 0.1% triton-X-100 at 16 hours. The lysates were plated on *Salmonella- Shigella* agar, and the corresponding CFU at 16 hours was determined. The percent abundance of cytosolic and vacuolar bacteria was determined by dividing the CFU from chloroquine treated set with chloroquine untreated set.


Percentageofcytosolicbacteria=[CFUat16hourswithchloroquine]/[CFUat16hourswithoutchloroquine]X100%

Percentageofvacuolarbacteria=(100‐percentageofcytosolicbacteria)%

### Confocal microscopy

For the immunofluorescence study, the infected RAW 264.7 or Caco-2 cells were fixed with 3.5% paraformaldehyde for 15 minutes. The cells were first incubated with specific primary antibody raised against *Salmonella* (Rabbit-raised anti- *Salmonella*), SseC/ SseD (Rabbit-raised anti- SseC/ SseD), mouse LAMP-1 (rat-raised anti-mouse LAMP-1), and mouse nitrotyrosine (mouse-raised anti-mouse nitrotyrosine), diluted in 2.5% BSA and 0.01% saponin (dilution- 1: 100, duration- 6 to 8 hours at 4°C temperature). This was followed by incubating the cells with appropriate secondary antibodies conjugated with fluorophores as mentioned in the figures (dilution- 1: 200, duration- 1 hour at room temperature). The coverslips were mounted with anti-fade reagent and fixed on a glass slide with transparent nail paint. Samples were imaged by confocal laser scanning microscopy (Zeiss LSM 710 or Zeiss LSM 880) using a 63X oil immersion objective lens. The images were analyzed with ZEN Black 2009 software provided by Zeiss. To determine the colocalization coefficient, the position of the scatterplot crosshairs corresponding to Cy3 (X-axis) and Alexa Fluor/ DyLight 488 (Y-axis) channels were adjusted according to the single labeled control samples. The colocalization of the bacteria (Cy3- red) with our protein of interest (LAMP-1/ nitrotyrosine/ SseC/ SseD) (Alex Fluor/ DyLight 488- green) was determined by quantifying the colocalization coefficient of Cy3 channel from the individual stack of an image.

### Griess assay to measure extracellular nitrite concentration

Extracellular nitrite from infected macrophage cells was measured using a protocol described earlier [79]. A standard curve has been prepared with 0, 3.13, 6.25, 12.5, 25, 50, and 100 μM concentrations of NaNO_2_. After adding the Griess reagent, the [OD]545nm of the standard solutions were measured. As described in the figure, culture supernatants were collected from RAW264.7 cells infected with bacteria. To 50 μL of culture supernatant, 50 μL of 1% sulphanilamide (made in 5% phosphoric acid), and 50 μL of 0.1% NED (N-1-naphthyl ethylene diamine dihydrochloride) was added and incubated for 10 minutes in darkness at room temperature. The [OD]_545nm_ was measured within 30 minutes of the appearance of a purple-colored product.

### Measurement of intracellular nitric oxide

The intracellular nitric oxide (NO) of infected macrophages was measured using cell membrane-permeable fluorescent nitric oxide probe 4, 5- diaminofluorescein diacetate (DAF2-DA) [80]. 16 hours post-infection, the infected macrophages were supplemented with a 5μM concentration of DAF2-DA and incubated for 30 minutes. The cells were washed with sterile PBS and acquired immediately for analysis by flow cytometry (BD FACSVerse by BD Biosciences-US) using a 491 nm excitation channel and 513 nm emission channel.

### Measurement of the activity of the *spiC* promoter

The activity of *spiC* promoter in STM (WT) and *ΔompA* was measured by altering a protocol described earlier [79]. Briefly, 1.5 mL of overnight grown stationary phase culture of STM (WT) and *ΔompA* carrying pHG86 *spiC-lacZ* construct were centrifuged at 6000 rpm for 10 minutes, and the pellet was resuspended in 500 μL of Z-buffer (Na2HPO4, 60 mM; NaH2PO4, 40 mM; KCl, 10 mM; MgSO4.7H2O, 1mM). The OD of the Z-buffer was measured at 600 nm after resuspension. The cells were permeabilized by adding 5 μL of 0.1% SDS and 20 μL of chloroform and incubated at room temperature for 5 minutes. 100 μL of 4 mg/ mL of o-nitrophenyl *β*-D galactopyranoside was added in the dark and incubated till the color appeared. The reaction was stopped using 250 μL 1 M Na_2_CO_3_. The reaction mixture was centrifuged at 6000 rpm for 10 minutes, and the OD of the supernatant was measured at 420 nm and 550 nm on flat bottom transparent 96 well plates. STM (WT) and *ΔompA* harboring promoter-less empty pHG86 *LacZ* construct were used as control. To measure the *spiC* promoter activity from the bacteria inside macrophages, the infected cells were lysed with 0.1% triton-X-100. The lysates collected from infected macrophages were centrifuged at 14,000 rpm for 30 minutes, and the pellet was resuspended in 500 μL of Z-buffer. The activity of the *spiC* promoter was measured in Miller Unit using the following formula

$$\begin{array}{l} Miller\;Unit\;(MU)=1000\;[OD_{420nm}-OD_{550nm}\;*\;1.75]/T*V*OD_{600nm}\\ {\mathrm{OD}}_{420\mathrm{nm}}=\mathrm{Absorbance}\;\mathrm{by}\;o‐\mathrm{nitrophenol}\;\mathrm{and}\;\mathrm{light}\;\mathrm{scattering}\;\mathrm{by}\;\mathrm{cell}\;\mathrm{debris}\\ OD_{550nm}=light\;scattering\;by\;cell\;debris\\ OD_{600nm}=bacterial\;cell\;density\;in\;the\;washed\;media\\ T=Time\;of\;reaction\;in\;\min utes\\ V=Volume\;of\;the\;culture\;in\;mL. \end{array}$$

### Measurement of the cytosolic acidification of the bacteria using BCECF-AM

The acidification of the cytosol of the bacteria in the presence of *in vitro* acidic stress was measured using a cell-permeable dual excitation ratiometric dye 2’,7’-Bis-(2-Carboxyethyl)-5-(and-6)-Carboxyfluorescein, Acetoxymethyl Ester (BCECF-AM). 4.5 X 10^7^ CFU of STM (WT), *ΔompA*, and *ΔompA*: pQE60-*ompA* from 12 hours old overnight grown stationary phase culture was resuspended in phosphate buffer of pH 5.5, 6, 6,5, and 7, respectively, and incubated for 2 hours in a shaker incubator at 37°C temperature. 30 minutes before the flow cytometry analysis, BCECF-AM was added to each tube to make the final concentration 20 μM. The bacterial cells were analyzed in flow cytometry (BD FACSVerse by BD Biosciences-US) using 405 nm and 488 nm excitation and 535 nm emission channel. The median fluorescence intensity (MFI) of the bacterial population at 488 nm and 405 nm was obtained from BD FACSuite software. The 488/ 405 ratio was determined to estimate the level of acidification of the bacterial cytosol.

### Measurement of extracellular H_2_O_2_ by phenol red assay

H_2_O_2_ produced by infected RAW264.7 cells was measured by modifying a protocol demonstrated earlier [81]. Briefly, two hours post-infection, infected RAW264.7 cells were supplemented with phenol red solution having potassium phosphate (0.01 M; pH 7.0), glucose (0.0055 M), NaCl (0.14 M), phenol red (0.2 g/ L), and HRPO (8.5 U/ mL; Sigma- Aldrich). 16 hours post-infection, the culture supernatant was collected and subjected to the [OD] measurement at 610 nm wavelength in TECAN 96 well microplate reader. In the presence of H_2_O_2_, horseradish peroxidase (HRPO) converts phenol red into a compound that has enhanced absorbance at 610 nm. The concentration of H_2_O_2_ produced by macrophages was measured using a standard curve of H_2_O_2_ in phenol red solution with known concentrations ranging from 0.5 to 5 μM.

### Measurement of intracellular ROS

The level of intracellular ROS from infected macrophages was measured using membrane-permeable redox-sensitive probe 2’,7’- dichlorodihydrofluorescein diacetate (H_2_DCFDA) [81]. Upon its oxidation by intracellular esterases, this non-fluorescent dye is converted into highly fluorescent 2’,7’- Dichlorofluorescein (H_2_DCF), which has emission at 492–495 nm and excitation at 517 to 527 nm. 16 hours post-infection, infected cells were supplemented with 10 μM of H_2_DCFDA, followed by incubating at 37°C temperature in the presence of 5% CO_2_ for 30 minutes. The cells were washed with sterile PBS and acquired immediately for analysis by flow cytometry (BD FACSVerse by BD Biosciences-US) using a 492 nm excitation channel and 517 nm emission channel.

### Sensitivity assay of bacteria against *in vitro* nitrosative and oxidative stress

The sensitivity of STM (WT) and *ΔompA* was tested against *in vitro* nitrosative and oxidative stress. H_2_O_2_ dissolved in PBS of pH 5.4 was used for creating *in vitro* oxidative stress. Acidified nitrite (NaNO_2_ in PBS of pH 5.4) alone and a combination of acidified nitrite and H_2_O_2_ were used to generate *in vitro* nitrosative stress [40]. Sensitivity was checked in both concentration and time-dependent manner. 10^8^ CFU of STM (WT) and *ΔompA* were added in varying concentrations of acidified nitrite and peroxide ranging from 200 μM to 5 mM and incubated for 12 hours. At the end of the incubation period, supernatants were plated to determine the inhibitory concentrations of nitrite, peroxide, and both. The concentration of acidified nitrite for the time-dependent study was 800μM. Aliquots were collected at 0, 3, 6, 9, and 12 hours post-inoculation to determine the CFU.

### Bacterial cell viability assay by resazurin

The viability of bacterial cells under acidified nitrite and peroxide treatment was measured by resazurin assay. Resazurin (color- blue) is reduced into resorufin (color- pink, excitation- 540 nm, and emission- 590 nm) by aerobic respiration of metabolically active cells. Bacteria treated with varying concentrations of acidified nitrite and peroxide were subjected to resazurin treatment (1 μg/mL) for 2 hours in a 37°C shaker incubator at 180 rpm. At the end of the incubation period, the fluorescence intensity was measured using TECAN 96 well microplate reader, and percent viability was calculated.

### Nitrite uptake assay

Nitrite consumption by bacteria was determined using a protocol described earlier [79]. Briefly, 10^8^ CFU of overnight grown bacterial cultures were inoculated in an uptake mixture consisting of 40 mM glucose, 80 mM MOPS-NaOH buffer (pH = 8.5), and nitrite (50–200 μM). The assay mixtures were kept in a 37°C shaker incubator. At indicated time points, 150 μL of suspension from each assay mixture was collected and subjected to Griess assay to determine the level of remaining nitrite.

### Examination of *in vitro* redox homeostasis of STM (WT) and *ΔompA* in response to acidified nitrite

Stationary phase cultures of STM (WT) and *ΔompA* harboring pQE60-Grx1-roGFP2 plasmid were sub-cultured in freshly prepared LB broth at 1: 33 ratios in the presence of appropriate antibiotic in a 37°C shaker incubator at 175 rpm. Once the [OD]_600 nm_ had reached 0.3 to 0.4, 500 μM of IPTG (Sigma-Aldrich) was added, and the cells were further grown at 30°C temperature for 10 to 12 hours. At the end of the incubation period, 4.5*10^7^ CFU of bacteria was subjected to the treatment of acidified nitrite for 15, 30, 45, and 60 minutes. At the end of every indicated time point, the cells were analyzed in flow cytometry (BD FACSVerse by BD Biosciences-US) using 405 nm and 488 nm excitation and 510 nm emission channel. The mean fluorescence intensity at 405 nm and 488 nm was obtained from the FITC positive (GFP expressing) population, and the 405/ 488 ratio was determined.

### Determination of outer membrane porosity of intracellular and extracellular bacteria by bisbenzimide

The outer membrane porosity of bacteria growing in acidic F media (KCl, (NH_4_)_2_SO_4_, K_2_SO_4_, KH_2_PO_4_, Cas-amino acids, glycerol, Tris-HCl, pH = 5.4) was measured using bisbenzimide (Sigma-Aldrich) by modifying a protocol as specified previously [82]. The bacterial strains were grown in F media for 12 hours. At the end of the incubation period, the culture supernatants were collected, and the [OD]_600 nm_ was adjusted to 0.1 with sterile PBS. 20 μL of bisbenzimide (10 μg/ mL) solution was added to 180 μL of culture supernatants in 96 well a microplate and further incubated for 10 minutes in a 37°C shaker incubator. The fluorescence intensity of DNA-bound bisbenzimide was measured in TECAN 96 well microplate reader using 346 nm excitation and 460 nm emission filter. To check the outer membrane porosity of intracellular bacteria, infected RAW264.7 macrophages were lysed with 0.1% Triton X-100. The lysate was centrifuged at 300g for 5 minutes to precipitate the eukaryotic cell debris. The supernatant was centrifuged at 5000 rpm for 20 minutes to precipitate the bacteria. To measure the fluorescence intensity, the bacterial pellet was resuspended in PBS and subjected to bisbenzimide treatment.

### Determination of bacterial membrane depolarization using DiBAC_4_

The bacterial outer membrane depolarization was measured using a fluorescent dye called bis-(1,3-dibutyl barbituric acid)-trimethylene oxonol (Invitrogen). Briefly, 4.5*10^7^ CFU of bacteria was incubated with 1μg/mL of DiBAC_4_ for 15 minutes in a 37°C shaker incubator. The DiBAC_4_ treated bacterial cells were further analyzed by flow cytometry (BD FACSVerse by BD Biosciences-US) to evaluate the change in membrane depolarization upon knocking out *ompA*. For determining the role of *ompA*, *ompC*, *ompD*, and *ompF* in bacterial outer membrane depolarization, 500 μM of IPTG was added to the cultures of STM (WT): pQE60, STM (WT): pQE60-*ompA*, STM (WT): pQE60-*ompC*, STM (WT): pQE60-*ompD*, and STM (WT): pQE60-*ompF* as mentioned earlier. At the end of the incubation period, the bacterial cells were subjected to the DiBAC_4_ treatment.

### Expression profiling of *ompC*, *ompD*, *ompF* in STM (WT), *ΔompA*, and complement strains growing in LB broth, acidic F media, and macrophages

Overnight cultures of STM (WT), *ΔompA*, & *ΔompA*: pQE60-*ompA* were inoculated in freshly prepared LB broth, F media at a 1: 100 ratio. The cells were grown in a 37°C shaker incubator at 180 rpm for 12 hours. RAW264.7 cells were infected with above mentioned bacterial strains at MOI 50 and incubated for 12 hours. At the end of the incubation period, RNA was isolated, cDNA was synthesized, and the expression of *ompC*, *ompD*, and *ompF* was checked.

### Live dead assay by propidium iodide

10^8^ CFU of overnight grown bacterial cultures were inoculated in 1 mM of acidified nitrite and incubated for 12 hours. After the incubation, the bacterial cells were subjected to propidium iodide (PI) (Sigma-Aldrich) (concentration- 1μg/ mL) treatment for 30 minutes at 37°C temperature. To estimate the percent viability, the PI-treated bacterial samples were analyzed by flow cytometry (BD FACSVerse by BD Biosciences-US).

### Animal survival assay

4–6 weeks old BALB/c and C57BL/6 mice housed in the specific-pathogen-free condition of the central animal facility of Indian Institute of Science, Bangalore, were used for all the *in vivo* infection and survival studies. The Institutional Animal Ethics Committee approved all the animal experiments, and the National Animal Care Guidelines were strictly followed. Two cohorts of twenty BALB/c and C57BL/6 mice were infected with stationary phase cultures of STM (WT) and *ΔompA* by oral gavaging at a lethal dose of 10^8^ CFU/ animal, respectively **(n = 10)**. The survival of mice was monitored for the next few days until all the mice infected with STM (WT) died. The survival was recorded, and the data was represented as percent survival.

### Determination of bacterial burden in different organs

Four cohorts of five 4–6 weeks old C57BL/6 mice were infected with STM (WT) and *ΔompA* by oral gavaging at 10^7^ CFU/ animal doses, respectively **(n = 5)**. Two of these cohorts infected with STM (WT) and *ΔompA* strains were further intraperitoneally injected with iNOS inhibitor aminoguanidine hydrochloride (AGH- 10mg/ kg of body weight) regularly for five days. The other two cohorts were treated with a placebo. Two cohorts of *iNOS*^*-/-*^ mice were orally infected with STM (WT) and *ΔompA* at 10^7^ CFU/ animal **(n = 5)**. On the 5^th^ day post-infection, all the mice were sacrificed, followed by isolation, weighing, and homogenizing of specific organs like- the liver, spleen, and MLN. The organ lysates were plated on *Salmonella Shigella* agar to determine the bacterial burden in different organs.

### Statistical analysis

Each assay has been repeated 2 to 5 times independently, as mentioned in the figure legends. The statistical analyses were done by unpaired two-tailed student’s *t*-test and one or two-way ANOVA followed by Dunnett or Tukey’s multiple comparisons test as indicated in the figure legends. *P* values below 0.05 were considered significant. The results are expressed as either mean ± SD or mean ± SEM. Data obtained from *in vivo* infection of mice were analyzed by Mann- Whitney *U* test. GraphPad Prism 8.4.3 (686) software was used to perform all the statistical analyses.

## Supporting information

S1 Fig *Salmonella* prefers OmpA over other larger porins while infecting macrophages. Transcript level expression profile of (A) *ompA*, (B) *ompC*, (C) *ompD*, and (D) *ompF* in STM- (WT) at indicated time points (3, 6, 9, 12 hours) in LB broth, acidic F media (pH = 5.4), and RAW264.7 murine macrophage cells (MOI = 50) by RT-qPCR (n = 3, N = 3). The relative expression of *ompA*, *ompC*, *ompD*, *and ompF* were represented in the log2 scale. The predicted structures of porins (A) OmpA, (B) OmpC, (C) OmpD, and (D) OmpF using the SWISS-MODEL protein structure homology-modeling server. Data are represented as mean ± SEM. *(P)* *< 0.05, *(P)* **< 0.005, *(P)* ***< 0.0005, *(P)* ****< 0.0001, ns = non-significant, (One-way ANOVA).
(TIF)

S2 Fig The growth kinetics of STM (WT) and *ΔompA* in LB broth. Studying the growth kinetics of STM (WT), *ΔompA*, *ΔompA*: pQE60-*ompA*, and *ΔompA*: pQE60 in LB broth culture at different time points (as indicated in the figure) (A) by measuring the absorbance at 600 nm (n = 3, N = 2) and (B) by plating the culture supernatant on LB agar (n = 2, N = 2).
(TIF)

S3 Fig Deletion of OmpA enhances the phagocytosis of *Salmonella* by macrophages. (A) Calculating the percent phagocytosis of STM (WT), *ΔompA*, *ΔompA*: pQE60-*ompA*, & *ΔompA*: pQE60 (MOI 10) by RAW 264.7 and PMA activated U937 cells (n = 3, N = 3 for RAW264.7 cells and n = 3, N = 2 for activated U937 cells). (B) The percent phagocytosis of STM (WT) and *ΔompA* either untreated or treated with 10% mouse complement sera (MOI of 50) by RAW264.7 cells (n = 3, N = 2). (C) Estimating the adhesion of STM (WT), *ΔompA*, *ΔompA*: pQE60-*ompA*, & *ΔompA*: pQE60 on the RAW 264.7 cells (MOI 50). 20 microscopic fields were analyzed. Adhesion was quantified by calculating the number of adherent bacteria/ total number of cells per field. Scale bar = 20μm (n = 20, N = 3). Data are represented as mean ± SEM. *(P)* *< 0.05, *(P)* **< 0.005, *(P)* ***< 0.0005, *(P)* ****< 0.0001, ns = non-significant, (One-way ANOVA).
(TIF)

S4 Fig The invasion of *Salmonella* in epithelial cells is significantly compromised in the absence of OmpA. (A) Calculating the percent invasion of STM (WT), *ΔompA*, *ΔompA*: pQE60-*ompA*, & *ΔompA*: pQE60 (MOI 10) by Caco-2 and HeLa cells (n = 3, N = 3). (B) Estimating the adhesion of STM (WT), *ΔompA*, *ΔompA*: pQE60-*ompA*, & *ΔompA*: pQE60 on the HeLa cells (MOI 50). 20 microscopic fields were analyzed. Adhesion was estimated by calculating the number of adherent bacteria/ total number of cells per field. Scale bar = 20μm (n = 20, N = 3). Data are represented as mean ± SEM. *(P)* *< 0.05, *(P)* **< 0.005, *(P)* ***< 0.0005, *(P)* ****< 0.0001, ns = non-significant, (One-way ANOVA).
(TIF)

S5 Fig STM *ΔompA* growing in *in vitro* growth media is deficient in expressing *sseC and sseD*. The expression of SPI-2 effector genes *sseC* (A-B) and *sseD* (C-D) in wild-type and OmpA deficient *Salmonella* growing in LB (A and C) and acidic F media (B and D) by RT-qPCR (n = 3, N = 3). Data are represented as mean ± SEM. *(P)* *< 0.05, *(P)* **< 0.005, *(P)* ***< 0.0005, *(P)* ****< 0.0001, ns = non-significant, (Student’s *t* test- unpaired).
(TIF)

S6 Fig OmpA does not play a significant role in protecting *Salmonella* from oxidative stress. (A) Representative dot plots (SSC-A vs. DCFDA) and histograms (Count vs. DCFDA) depicting the level of intracellular reactive oxygen species (ROS) in RAW 264.7 cells infected with STM (WT), *ΔompA*, and *ΔompA*: pQE60-*ompA* (MOI 10). (B) The percent population of DACFDA positive RAW264.7 cells, (n = 4, N = 3). (C) Quantifying the level of extracellular ROS from the culture supernatant of RAW264.7 cells infected with STM (WT), *ΔompA*, *ΔompA*: pQE60-*ompA*, *ΔompA*: pQE60, & PFA fixed dead bacteria (MOI 10) (n = 3, N = 2). The *in vitro* sensitivity of STM (WT) and *ΔompA* in the presence of (D) H_2_O_2_, (E) acidified nitrite, and (F) NaNO_2_ and H_2_O_2_ combined by calculating the CFU (N = 3) and resazurin test (n = 3, N = 3). Data are represented as mean ± SEM. *(P)* *< 0.05, *(P)* **< 0.005, *(P)* ***< 0.0005, *(P)* ****< 0.0001, ns = non-significant, (One-way ANOVA in B, C and unpaired student’s *t* test in D, E, F).
(TIF)

S7 Fig Complementing STM *ΔompA* with *spiC-lacZ* construct restored the *spiC* promoter activity in *in vitro* growth media. (A) Measuring the activity of *spiC* promoter in STM (WT) and *ΔompA* growing in acidic F media and LB broth culture (n = 6). (B) Studying the activity of *spiC* promoter in STM (WT) and *ΔompA* proliferating intracellularly in RAW264.7 cells (MOI = 50) at 12 hours post-infection. Data are represented as mean ± SEM (n = 5, N = 2). *(P)* *< 0.05, *(P)* **< 0.005, *(P)* ***< 0.0005, *(P)* ****< 0.0001, ns = non-significant, (One-way ANOVA).
(TIF)

S8 Fig STM *ΔompA* induces reactive nitrogen intermediates in macrophages due to reduced expression of SPI-2 effectors. (A) Fold proliferation of STM (WT), *ΔompA*, *ΔsifA*, *ΔompAΔsifA*, *ΔssaV* and *ΔompAΔssaV* (MOI 10) in RAW264.7 cells (n = 3, N = 2). (B) Representative dot plots (SSC-A vs. DAF-2 DA) and histograms (Count vs. DAF-2 DA) of RAW264.7 cells infected with STM (WT), *ΔompA*, *ΔsifA*, *ΔompAΔsifA*, *ΔssaV* and *ΔompAΔssaV* (MOI 10) to estimate the level of intracellular nitric oxide (NO) using DAF-2 DA (5 μM). The percent population of DAF-2 DA positive cells was represented in a vertical bar graph (n≥3, N = 2). Data are represented as mean ± SEM. *(P)* *< 0.05, *(P)* **< 0.005, *(P)* ***< 0.0005, *(P)* ****< 0.0001, ns = non-significant, (One-way ANOVA).
(TIF)

S9 Fig STM *ΔompA* induced nitrosative burst in Caco-2 cells is equivalent to the wild-type *Salmonella*. (A) Representative dot plots (SSC-A vs. DAF-2 DA) and histograms (Count vs. DAF-2 DA) of Caco-2 cells infected with STM (WT), *ΔompA*, and *ΔompA*: pQE60-*ompA* (MOI 10) to estimate the level of intracellular nitric oxide (NO) using DAF-2 DA (5 μM). A vertical bar graph represented the percent population of DAF-2 DA positive Caco-2 cells. Data are represented as mean ± SEM (n = 3, N = 2). **ns = non-significant, (One-way ANOVA)**.
(TIF)

S10 Fig Over-expression of *ompF* in wild-type *Salmonella* enhances the outer membrane porosity. The representative dot plots (SSC-A vs. DiBAC_4_) and histograms (Count vs. DiBAC_4_) measuring the outer membrane porosity of STM (WT), STM (WT): pQE60, STM (WT): pQE60-*ompA*, STM (WT): pQE60-*ompC*, STM (WT): pQE60-*ompD*, and STM (WT): pQE60-*ompF* in (A) acidic F media and (C) LB broth with 500 μM of IPTG using DiBAC_4_ (final concentration- 1 μg/ mL). The percent population of DiBAC_4_ positive cells in acidic F media (B) and LB broth culture (D) has been represented in vertical bar graphs. Data are represented as mean ± SEM (n = 6, N = 3 for B)/ SD (n = 6 for D). *(P)* *< 0.05, *(P)* **< 0.005, *(P)* ***< 0.0005, *(P)* ****< 0.0001, ns = non-significant, (One-way ANOVA)
(TIF)

S11 Fig The deletion of *ompC*, *ompD*, and *ompF* does not hamper the viability of *Salmonella* against intracellular and extracellular nitrosative stress. (A) Representative images of RAW264.7 cells infected with STM (WT), *ΔompA*, *ΔompC*, *ΔompD*, and *ΔompF* (MOI 20). Quantification of nitrotyrosine recruitment on STM (WT), *ΔompA*, *ΔompC*, *ΔompD*, and *ΔompF* has been represented in a vertical bar graph. (n≥60, N = 3). Scale bar = 5μm. (B) Checking the *in vitro* sensitivity of STM (WT), *ΔompA*, *ΔompC*, *ΔompD*, and *ΔompF* in the presence of acidified nitrite (N = 3). (C) *In vitro* nitrite uptake assay of STM (WT), *ΔompA*, *ΔompC*, *ΔompD*, *ΔompF* & PFA fixed dead bacteria (n = 3, N = 3). All the data are represented as mean ± SEM. *(P)* *< 0.05, *(P)* **< 0.005, *(P)* ***< 0.0005, *(P)* ****< 0.0001, ns = non-significant, (One-way ANOVA in A and unpaired student’s *t* test in B, C).
(TIF)

S12 Fig Overexpression of *ompF* enhances the susceptibility of wild-type *Salmonella* to *in vitro* nitrosative stress. (A) Representative dot plots (SSC-A vs. DiBAC_4_) and histograms (Count vs. DiBAC_4_) measuring the *in vitro* viability of STM (WT), STM (WT): pQE60, STM (WT): pQE60-*ompA*, STM (WT): pQE60-*ompC*, STM (WT): pQE60-*ompD*, and STM (WT): pQE60-*ompF* using propidium iodide (final concentration- 1 μg/ mL) in the presence of acidified nitrite (PBS of pH = 5.4 and 1 mM NaNO_2_). Percent population of propidium iodide positive cells from (B) acidified PBS and (C) acidified nitrite have been represented here in the form of a vertical bar graph. Data are represented as mean ± SEM (n = 3, N = 2 for B and n = 8, N = 2 for C). Measuring the *in vitro* viability of STM (WT), STM (WT): pQE60, STM (WT): pQE60-*ompA*, STM (WT): pQE60-*ompC*, STM (WT): pQE60-*ompD*, and STM (WT): pQE60-*ompF* in acidified PBS (D and F) and acidified nitrite (E and F) using resazurin (final concentration- 0.002 mg/ mL). Data are represented as mean ± SD (n = 6 for D and n = 8 for E). *(P)* *< 0.05, *(P)* **< 0.005, *(P)* ***< 0.0005, *(P)* ****< 0.0001, ns = non-significant, (One-way ANOVA).
(TIF)
