# Supplementary figures and images for: Salmonella Typhimurium outer membrane protein A (OmpA) renders protection from nitrosative stress of macrophages by maintaining the stability of bacterial outer membrane
Source: PLoS Pathog. 2022 Aug 15;18(8):e1010708. doi: 10.1371/journal.ppat.1010708 (PMC9410544; doi:10.1371/journal.ppat.1010708)

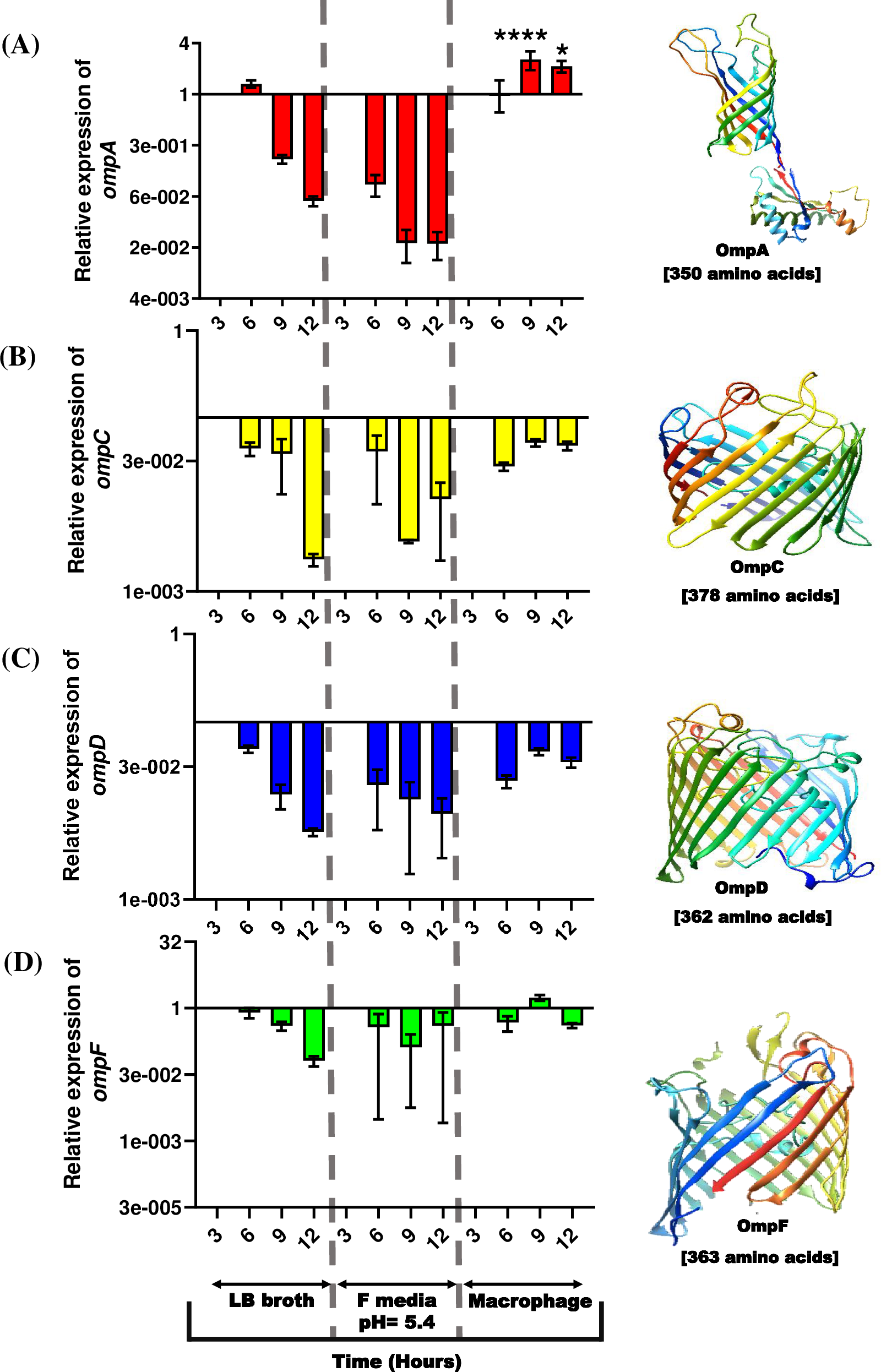

Supplement: S1 Fig — Transcript level expression profile of (A) ompA, (B) ompC, (C) ompD, and (D) ompF in STM- (WT) at indicated time points (3, 6, 9, 12 hours) in LB broth, acidic F media (pH = 5.4), and RAW264.7 murine macrophage cells (MOI = 50) by RT-qPCR (n = 3, N = 3). The relative expression of ompA, ompC, ompD, and ompF were represented in the log2 scale. The predicted structures of porins (A) OmpA, (B) OmpC, (C) OmpD, and (D) OmpF using the SWISS-MODEL protein structure homology-modeling server. Data are represented as mean ± SEM. (P) *< 0.05, (P) **< 0.005, (P) ***< 0.0005, (P) ****< 0.0001, ns = non-significant, (One-way ANOVA). (TIF) [file ppat.1010708.s001.tif]

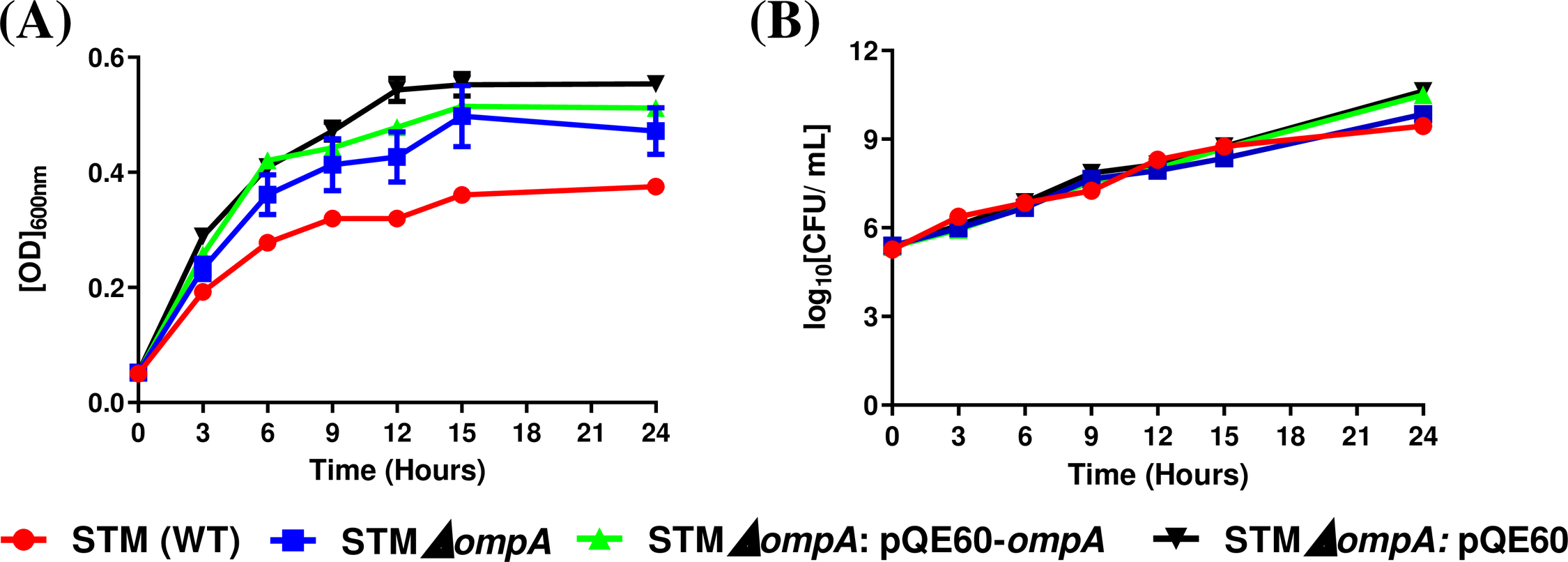

Supplement: S2 Fig — Studying the growth kinetics of STM (WT), ΔompA, ΔompA: pQE60-ompA, and ΔompA: pQE60 in LB broth culture at different time points (as indicated in the figure) (A) by measuring the absorbance at 600 nm (n = 3, N = 2) and (B) by plating the culture supernatant on LB agar (n = 2, N = 2). (TIF) [file ppat.1010708.s002.tif]

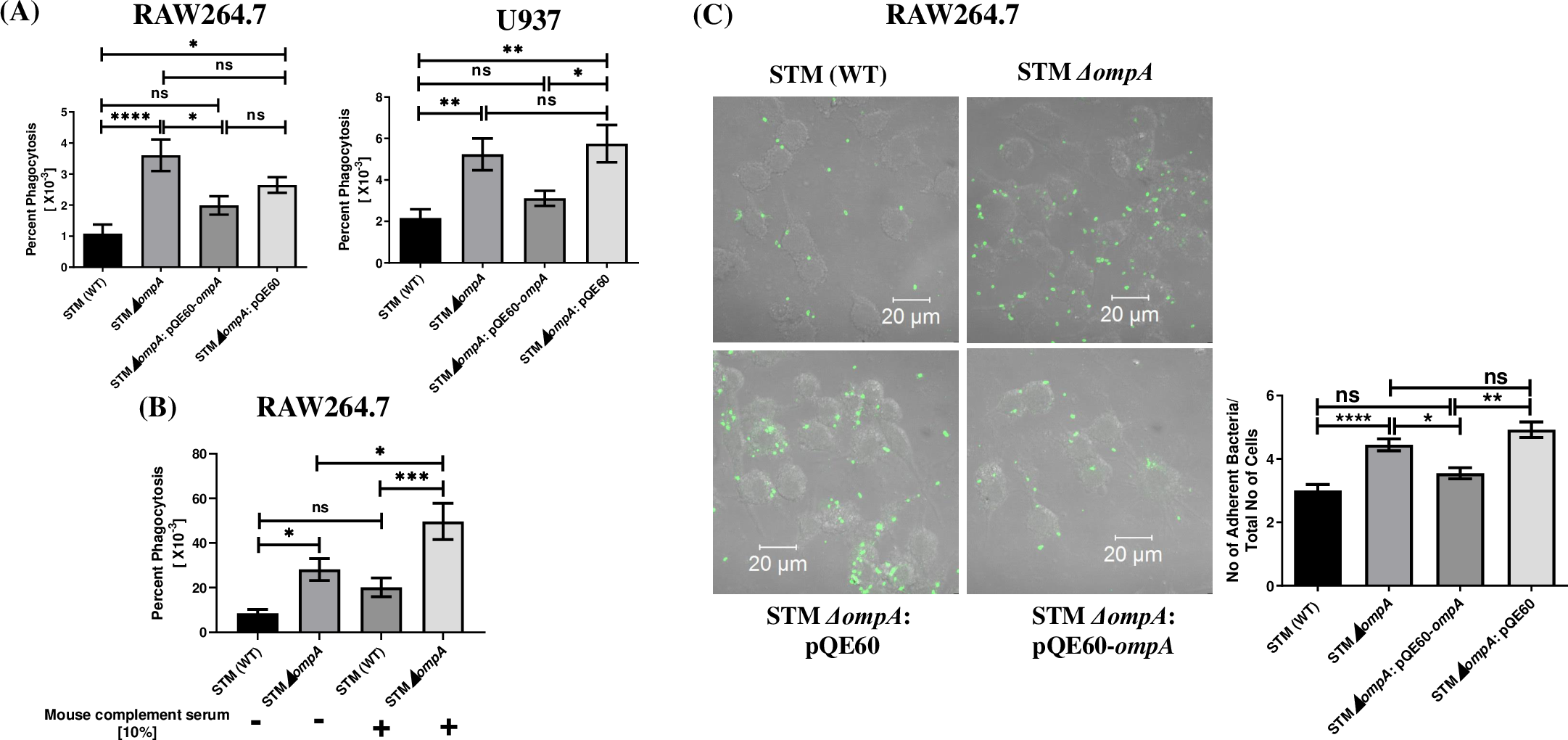

Supplement: S3 Fig — (A) Calculating the percent phagocytosis of STM (WT), ΔompA, ΔompA: pQE60-ompA, & ΔompA: pQE60 (MOI 10) by RAW 264.7 and PMA activated U937 cells (n = 3, N = 3 for RAW264.7 cells and n = 3, N = 2 for activated U937 cells). (B) The percent phagocytosis of STM (WT) and ΔompA either untreated or treated with 10% mouse complement sera (MOI of 50) by RAW264.7 cells (n = 3, N = 2). (C) Estimating the adhesion of STM (WT), ΔompA, ΔompA: pQE60-ompA, & ΔompA: pQE60 on the RAW 264.7 cells (MOI 50). 20 microscopic fields were analyzed. Adhesion was quantified by calculating the number of adherent bacteria/ total number of cells per field. Scale bar = 20μm (n = 20, N = 3). Data are represented as mean ± SEM. (P) *< 0.05, (P) **< 0.005, (P) ***< 0.0005, (P) ****< 0.0001, ns = non-significant, (One-way ANOVA). (TIF) [file ppat.1010708.s003.tif]

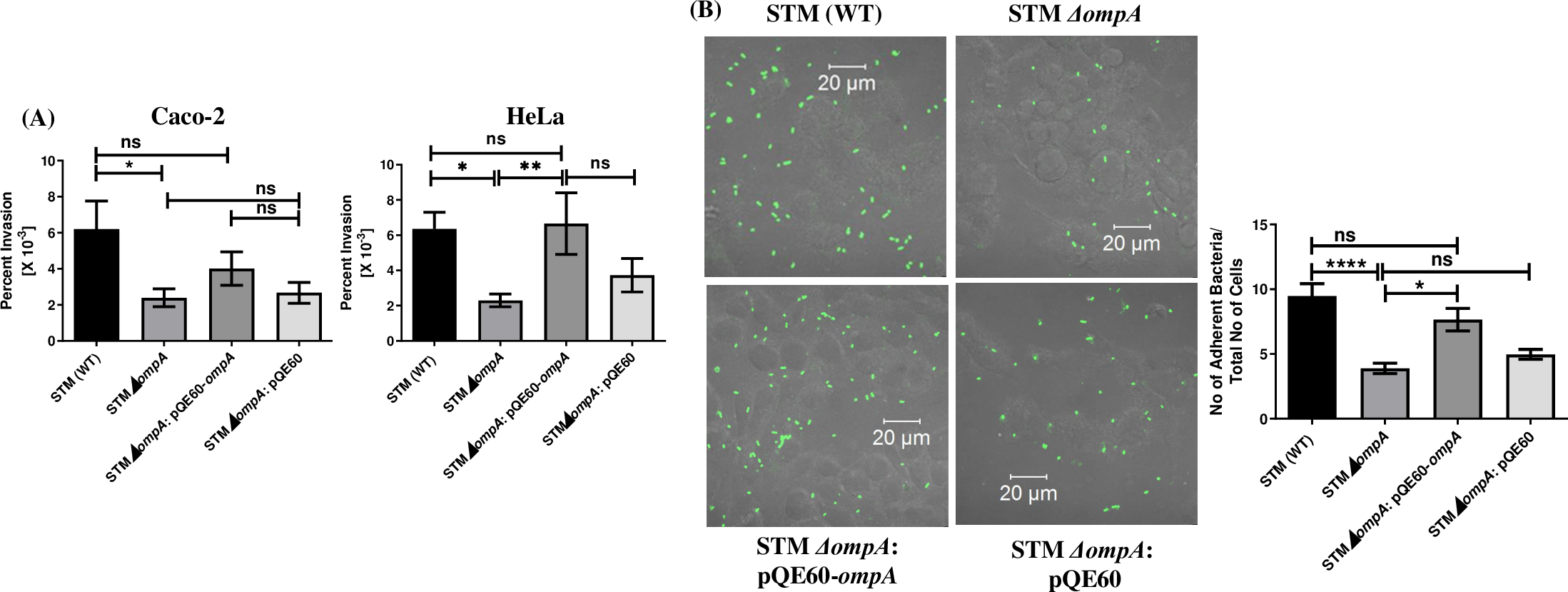

Supplement: S4 Fig — (A) Calculating the percent invasion of STM (WT), ΔompA, ΔompA: pQE60-ompA, & ΔompA: pQE60 (MOI 10) by Caco-2 and HeLa cells (n = 3, N = 3). (B) Estimating the adhesion of STM (WT), ΔompA, ΔompA: pQE60-ompA, & ΔompA: pQE60 on the HeLa cells (MOI 50). 20 microscopic fields were analyzed. Adhesion was estimated by calculating the number of adherent bacteria/ total number of cells per field. Scale bar = 20μm (n = 20, N = 3). Data are represented as mean ± SEM. (P) *< 0.05, (P) **< 0.005, (P) ***< 0.0005, (P) ****< 0.0001, ns = non-significant, (One-way ANOVA). (TIF) [file ppat.1010708.s004.tif]

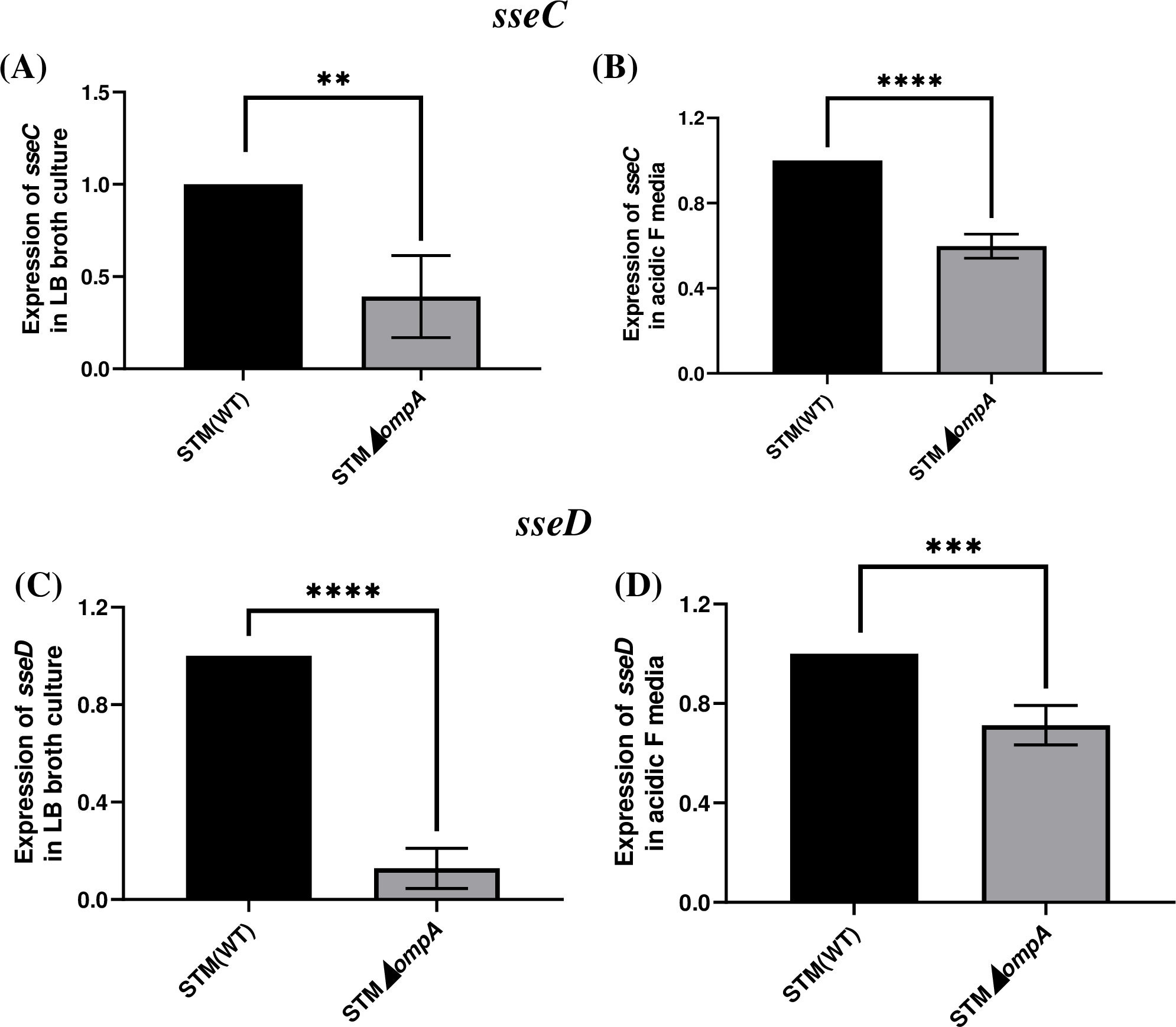

Supplement: S5 Fig — The expression of SPI-2 effector genes sseC (A-B) and sseD (C-D) in wild-type and OmpA deficient Salmonella growing in LB (A and C) and acidic F media (B and D) by RT-qPCR (n = 3, N = 3). Data are represented as mean ± SEM. (P) *< 0.05, (P) **< 0.005, (P) ***< 0.0005, (P) ****< 0.0001, ns = non-significant, (Student’s t test- unpaired). (TIF) [file ppat.1010708.s005.tif]

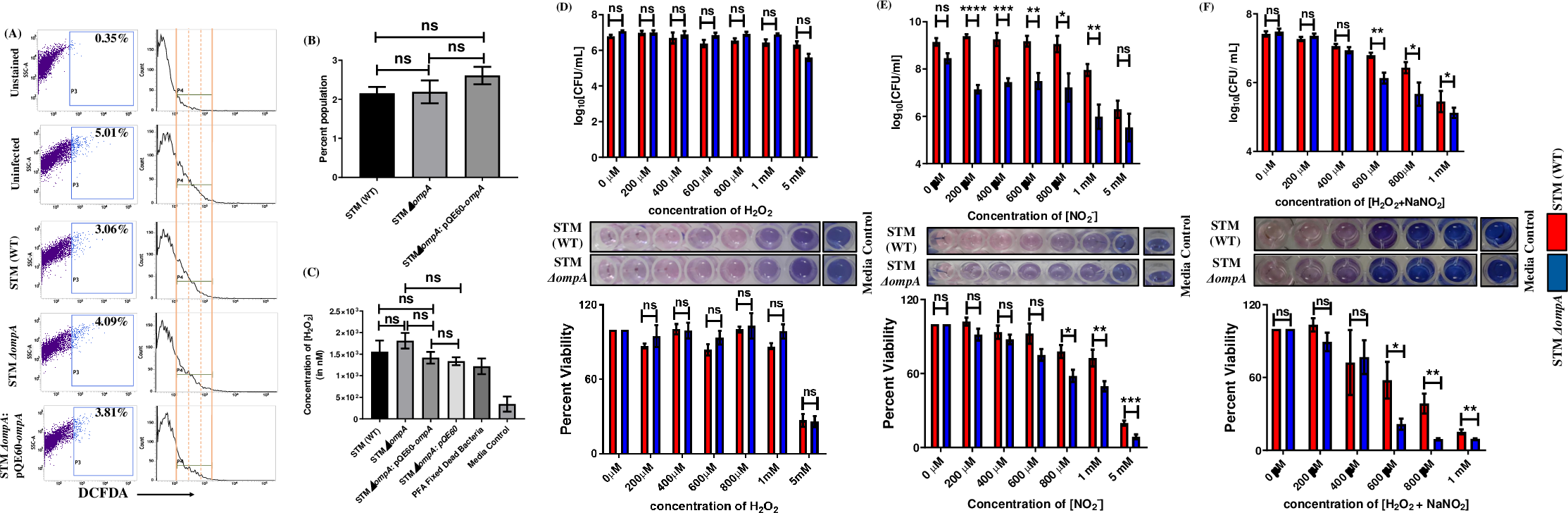

Supplement: S6 Fig — (A) Representative dot plots (SSC-A vs. DCFDA) and histograms (Count vs. DCFDA) depicting the level of intracellular reactive oxygen species (ROS) in RAW 264.7 cells infected with STM (WT), ΔompA, and ΔompA: pQE60-ompA (MOI 10). (B) The percent population of DACFDA positive RAW264.7 cells, (n = 4, N = 3). (C) Quantifying the level of extracellular ROS from the culture supernatant of RAW264.7 cells infected with STM (WT), ΔompA, ΔompA: pQE60-ompA, ΔompA: pQE60, & PFA fixed dead bacteria (MOI 10) (n = 3, N = 2). The in vitro sensitivity of STM (WT) and ΔompA in the presence of (D) H2O2, (E) acidified nitrite, and (F) NaNO2 and H2O2 combined by calculating the CFU (N = 3) and resazurin test (n = 3, N = 3). Data are represented as mean ± SEM. (P) *< 0.05, (P) **< 0.005, (P) ***< 0.0005, (P) ****< 0.0001, ns = non-significant, (One-way ANOVA in B, C and unpaired student’s t test in D, E, F). (TIF) [file ppat.1010708.s006.tif]

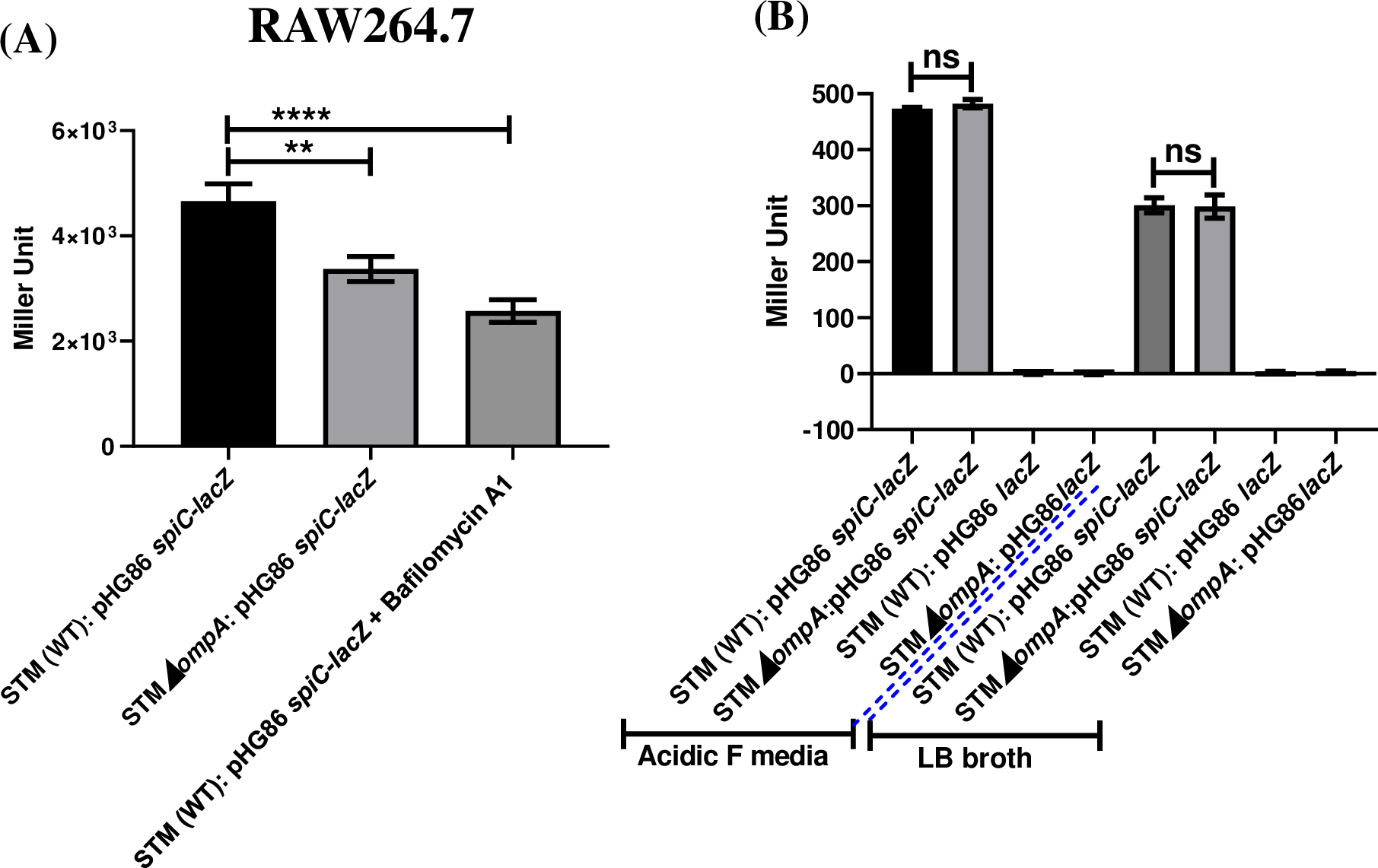

Supplement: S7 Fig — (A) Measuring the activity of spiC promoter in STM (WT) and ΔompA growing in acidic F media and LB broth culture (n = 6). (B) Studying the activity of spiC promoter in STM (WT) and ΔompA proliferating intracellularly in RAW264.7 cells (MOI = 50) at 12 hours post-infection. Data are represented as mean ± SEM (n = 5, N = 2). (P) *< 0.05, (P) **< 0.005, (P) ***< 0.0005, (P) ****< 0.0001, ns = non-significant, (One-way ANOVA). (TIF) [file ppat.1010708.s007.tif]

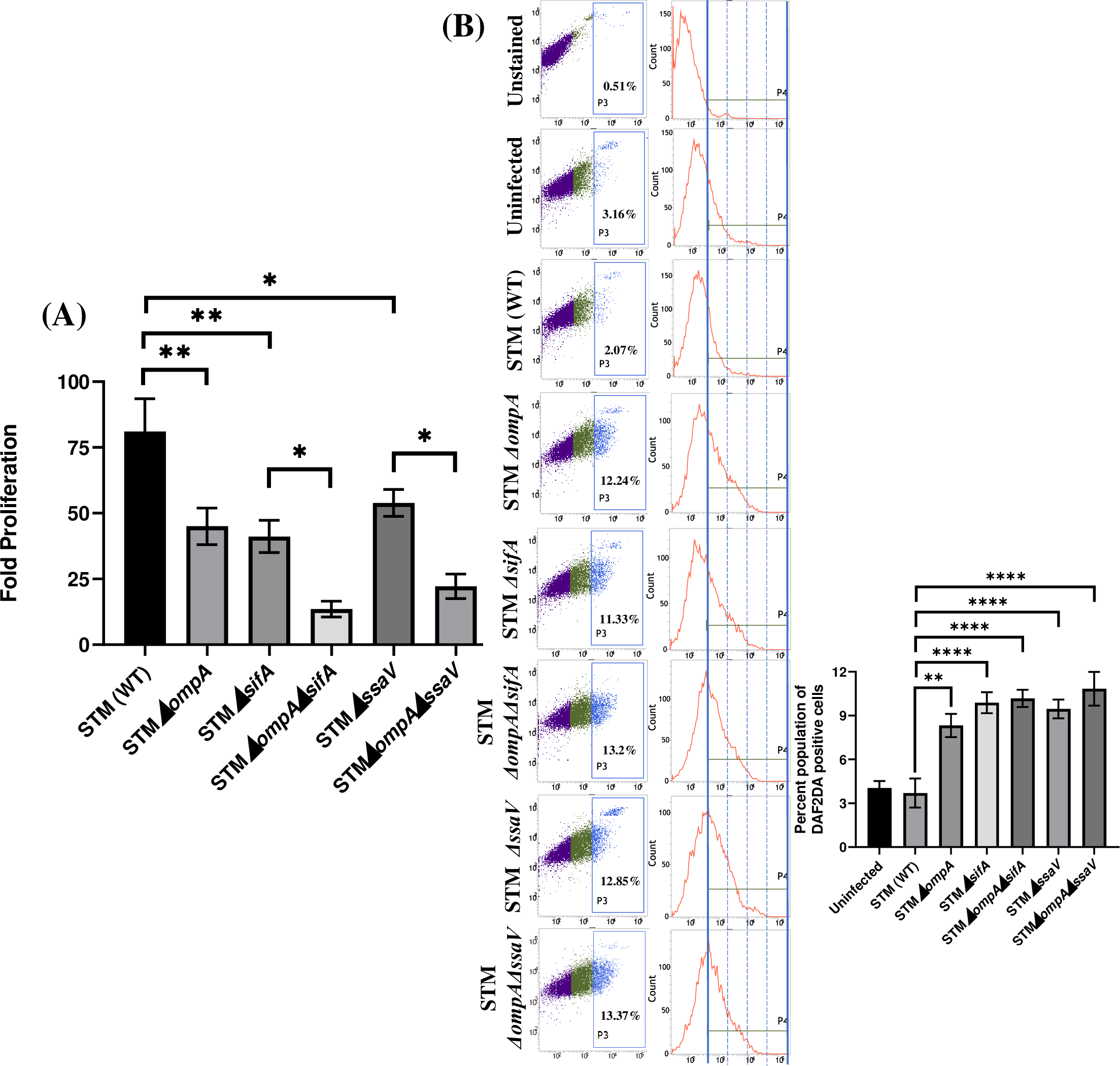

Supplement: S8 Fig — (A) Fold proliferation of STM (WT), ΔompA, ΔsifA, ΔompAΔsifA, ΔssaV and ΔompAΔssaV (MOI 10) in RAW264.7 cells (n = 3, N = 2). (B) Representative dot plots (SSC-A vs. DAF-2 DA) and histograms (Count vs. DAF-2 DA) of RAW264.7 cells infected with STM (WT), ΔompA, ΔsifA, ΔompAΔsifA, ΔssaV and ΔompAΔssaV (MOI 10) to estimate the level of intracellular nitric oxide (NO) using DAF-2 DA (5 μM). The percent population of DAF-2 DA positive cells was represented in a vertical bar graph (n≥3, N = 2). Data are represented as mean ± SEM. (P) *< 0.05, (P) **< 0.005, (P) ***< 0.0005, (P) ****< 0.0001, ns = non-significant, (One-way ANOVA). (TIF) [file ppat.1010708.s008.tif]

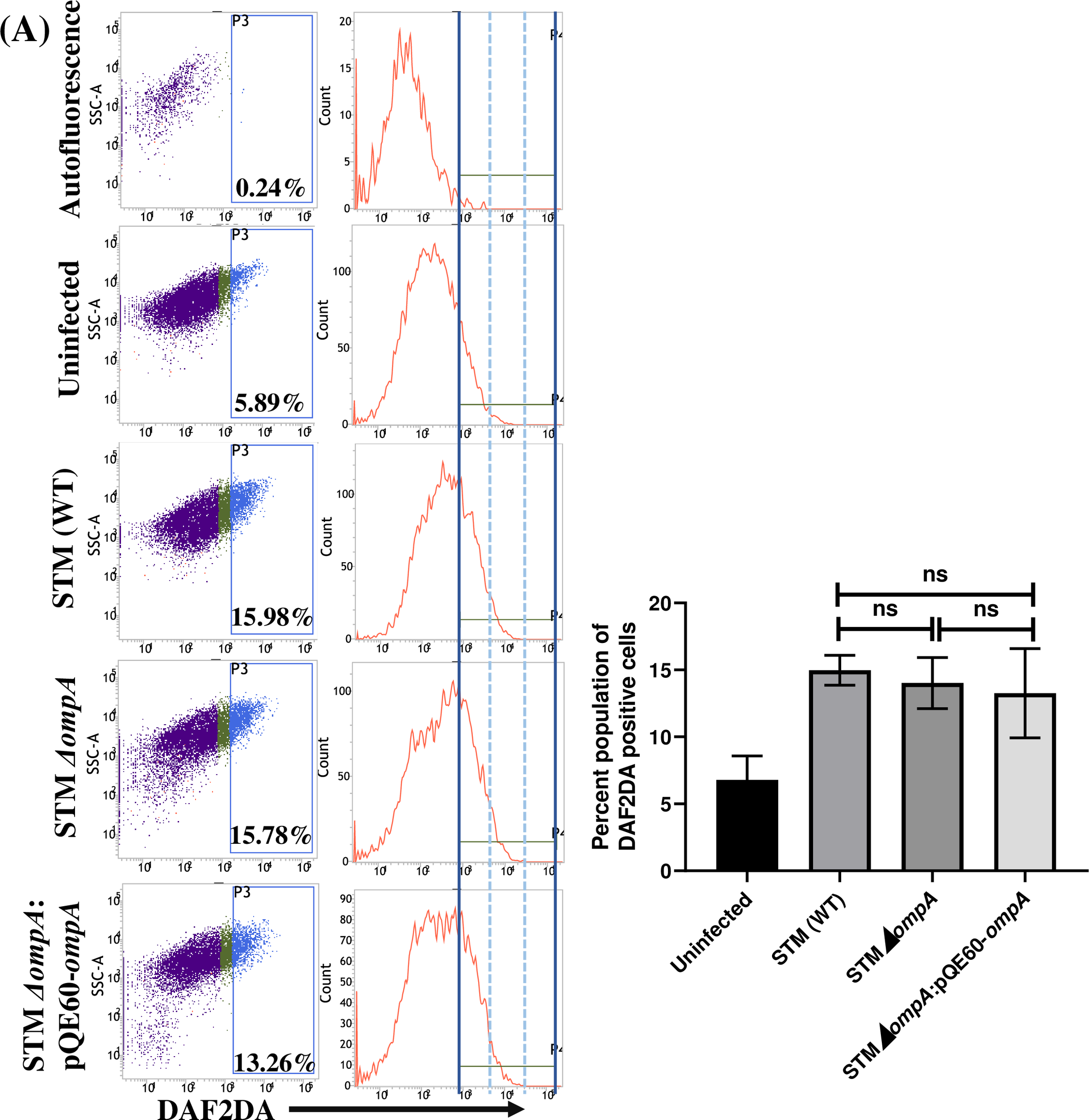

Supplement: S9 Fig — (A) Representative dot plots (SSC-A vs. DAF-2 DA) and histograms (Count vs. DAF-2 DA) of Caco-2 cells infected with STM (WT), ΔompA, and ΔompA: pQE60-ompA (MOI 10) to estimate the level of intracellular nitric oxide (NO) using DAF-2 DA (5 μM). A vertical bar graph represented the percent population of DAF-2 DA positive Caco-2 cells. Data are represented as mean ± SEM (n = 3, N = 2). ns = non-significant, (One-way ANOVA). (TIF) [file ppat.1010708.s009.tif]

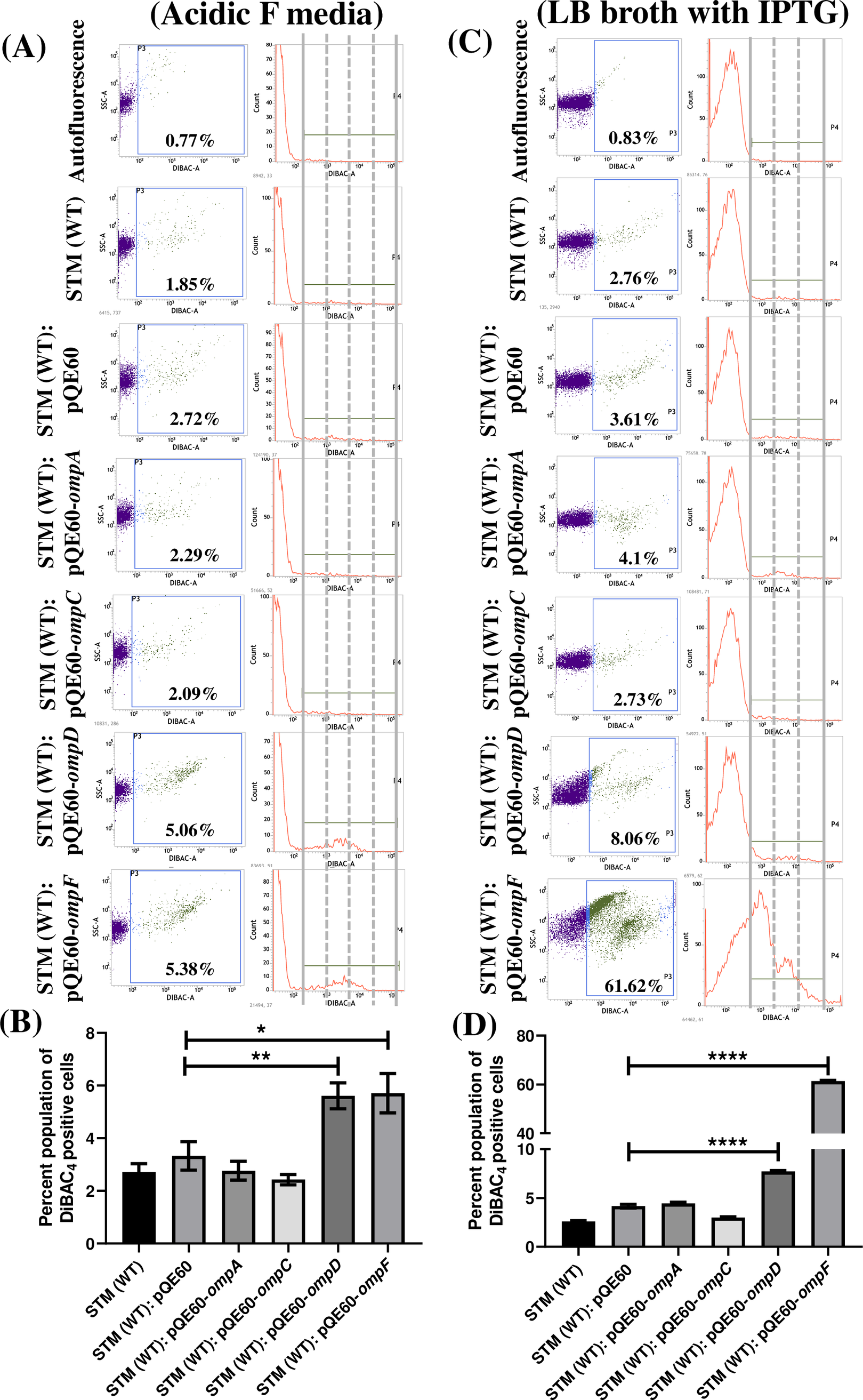

Supplement: S10 Fig — The representative dot plots (SSC-A vs. DiBAC4) and histograms (Count vs. DiBAC4) measuring the outer membrane porosity of STM (WT), STM (WT): pQE60, STM (WT): pQE60-ompA, STM (WT): pQE60-ompC, STM (WT): pQE60-ompD, and STM (WT): pQE60-ompF in (A) acidic F media and (C) LB broth with 500 μM of IPTG using DiBAC4 (final concentration- 1 μg/ mL). The percent population of DiBAC4 positive cells in acidic F media (B) and LB broth culture (D) has been represented in vertical bar graphs. Data are represented as mean ± SEM (n = 6, N = 3 for B)/ SD (n = 6 for D). (P) *< 0.05, (P) **< 0.005, (P) ***< 0.0005, (P) ****< 0.0001, ns = non-significant, (One-way ANOVA) (TIF) [file ppat.1010708.s010.tif]

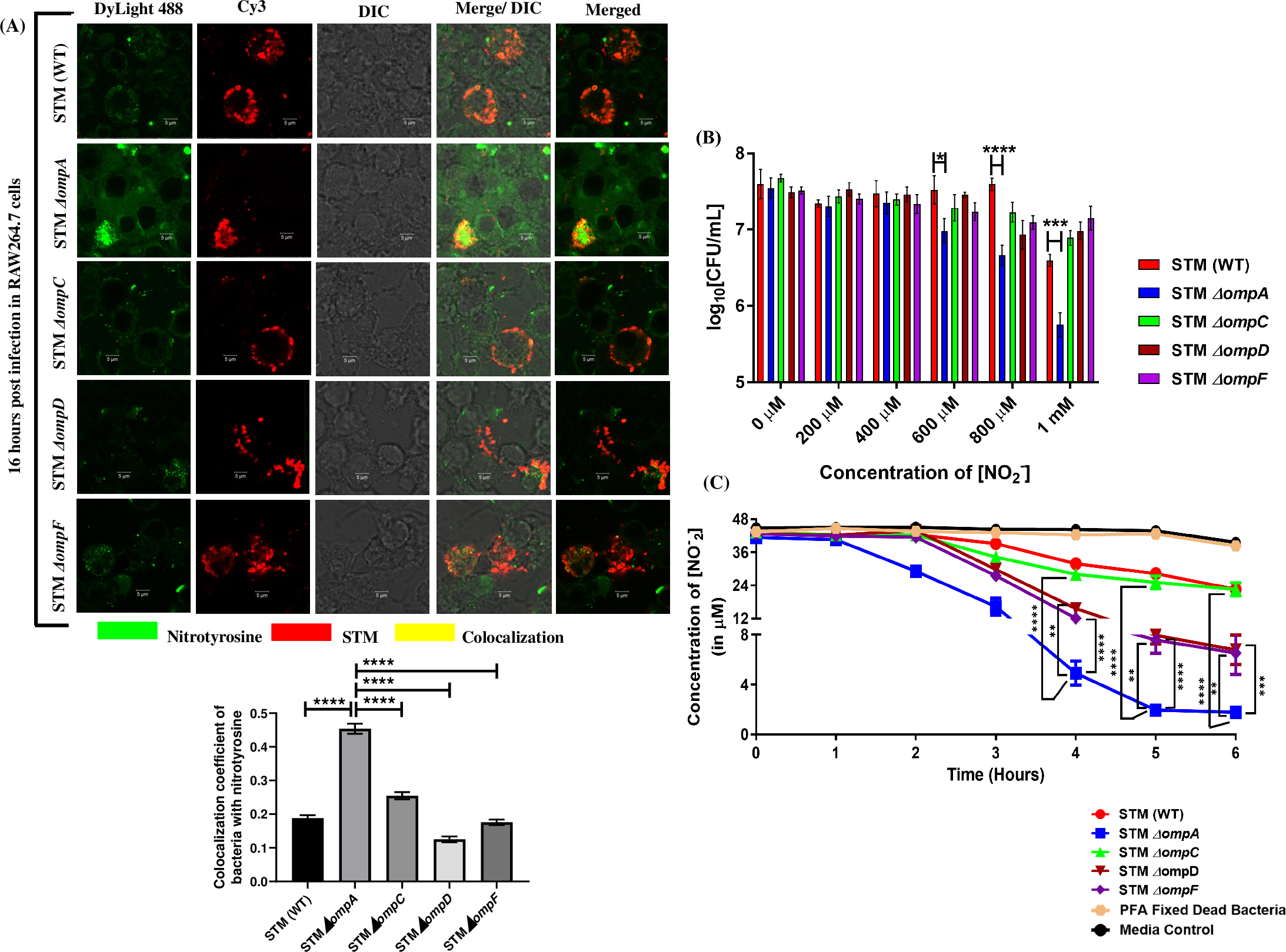

Supplement: S11 Fig — (A) Representative images of RAW264.7 cells infected with STM (WT), ΔompA, ΔompC, ΔompD, and ΔompF (MOI 20). Quantification of nitrotyrosine recruitment on STM (WT), ΔompA, ΔompC, ΔompD, and ΔompF has been represented in a vertical bar graph. (n≥60, N = 3). Scale bar = 5μm. (B) Checking the in vitro sensitivity of STM (WT), ΔompA, ΔompC, ΔompD, and ΔompF in the presence of acidified nitrite (N = 3). (C) In vitro nitrite uptake assay of STM (WT), ΔompA, ΔompC, ΔompD, ΔompF & PFA fixed dead bacteria (n = 3, N = 3). All the data are represented as mean ± SEM. (P) *< 0.05, (P) **< 0.005, (P) ***< 0.0005, (P) ****< 0.0001, ns = non-significant, (One-way ANOVA in A and unpaired student’s t test in B, C). (TIF) [file ppat.1010708.s011.tif]

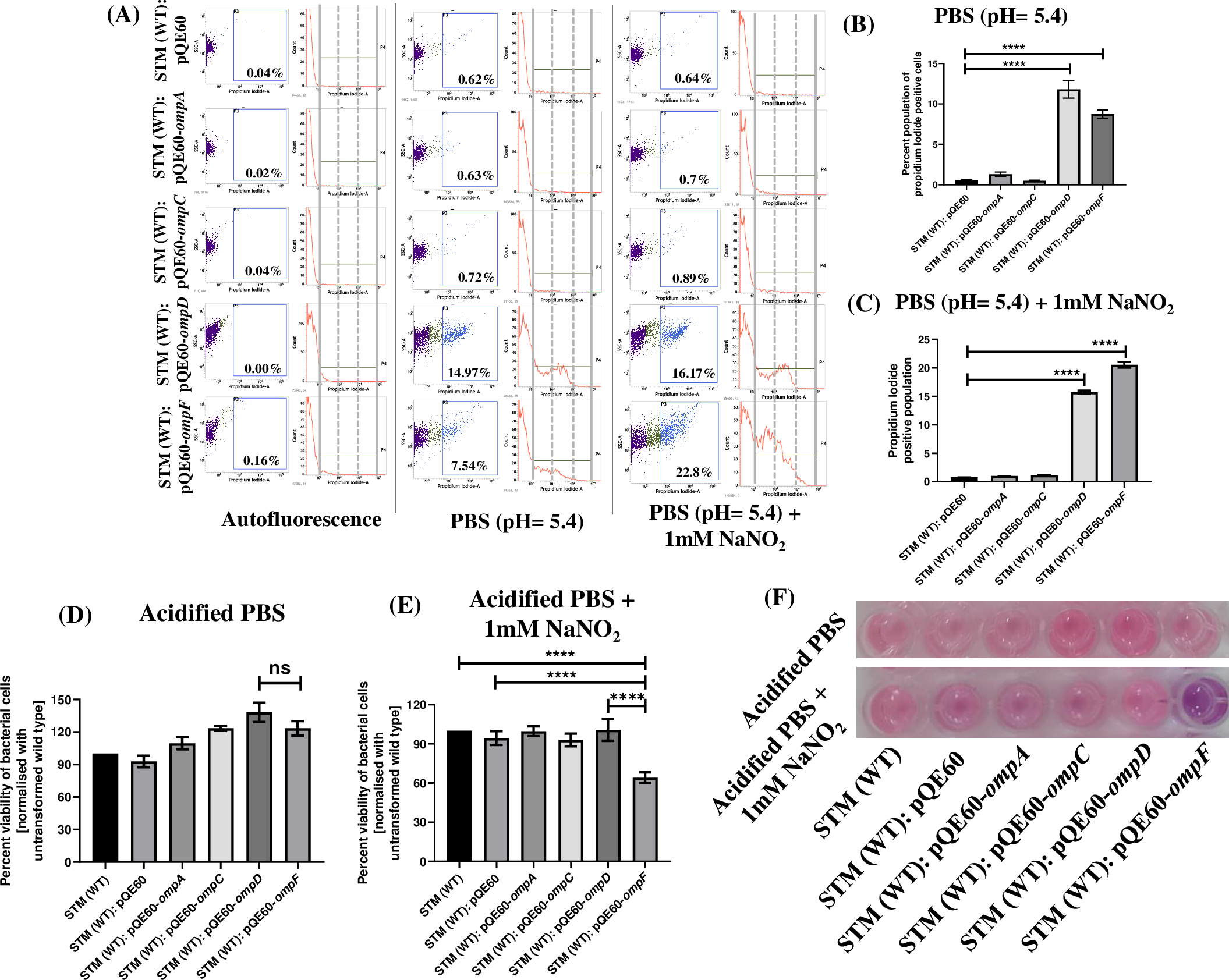

Supplement: S12 Fig — (A) Representative dot plots (SSC-A vs. DiBAC4) and histograms (Count vs. DiBAC4) measuring the in vitro viability of STM (WT), STM (WT): pQE60, STM (WT): pQE60-ompA, STM (WT): pQE60-ompC, STM (WT): pQE60-ompD, and STM (WT): pQE60-ompF using propidium iodide (final concentration- 1 μg/ mL) in the presence of acidified nitrite (PBS of pH = 5.4 and 1 mM NaNO2). Percent population of propidium iodide positive cells from (B) acidified PBS and (C) acidified nitrite have been represented here in the form of a vertical bar graph. Data are represented as mean ± SEM (n = 3, N = 2 for B and n = 8, N = 2 for C). Measuring the in vitro viability of STM (WT), STM (WT): pQE60, STM (WT): pQE60-ompA, STM (WT): pQE60-ompC, STM (WT): pQE60-ompD, and STM (WT): pQE60-ompF in acidified PBS (D and F) and acidified nitrite (E and F) using resazurin (final concentration- 0.002 mg/ mL). Data are represented as mean ± SD (n = 6 for D and n = 8 for E). (P) *< 0.05, (P) **< 0.005, (P) ***< 0.0005, (P) ****< 0.0001, ns = non-significant, (One-way ANOVA). (TIF) [file ppat.1010708.s012.tif]
